# Comparing caloric and self-experienced dizziness symptoms: diagnostic value and implications in functional dizziness

**DOI:** 10.1007/s00415-025-13334-3

**Published:** 2025-08-30

**Authors:** Jörn K. Pomper, Saskia Rabe, Uwe Ilg, Stephan Wolpert

**Affiliations:** 1 Tübingen Center for Dizziness and Balance Disorders, University of Tübingen, Tübingen, Germany; 2 Department of Neurology & Stroke, Hertie Institute for Clinical Brain Research, University of Tübingen, Tübingen, Germany; 3 Hertie Institute for Clinical Brain Research, University of Tübingen, Tübingen, Germany; 4 Department of Otolaryngology, Head and Neck Surgery, University of Tübingen, Tübingen, Germany

**Keywords:** Caloric stimulation, Dizziness questionnaire, Vertigo, Motion perception, Vestibular disorders, Persistent postural–perceptual dizziness

## Abstract

**Background:**

The diagnostic value of dizziness symptom quality is limited by variability in patient self-reports. Comparing it to the experience during standardized caloric stimulation could help control for individual differences in dizziness experience and reporting. As a nonphysiological stimulus, caloric testing may serve as a proxy for acute peripheral vestibular disorder. We hypothesized that dizziness of peripheral origin would be perceived as more similar to caloric stimulation than nonperipheral dizziness.

**Methods:**

Patients with peripheral (*n* = 49) and nonperipheral dizziness (*n* = 34) were compared. Using newly developed questionnaires, participants rated the intensity of 30 symptoms during both dizziness and caloric stimulation, compared symptom intensity between the two, and rated overall similarity.

**Results:**

Peripheral patients did not perceive caloric stimulation as more similar to their symptoms than nonperipheral patients. This also held for the functional dizziness subgroup (*n* = 14). However, exploratory analyses suggest symptom-specific group differences based on the directional intensity difference. For example, peripheral patients reported stronger spinning during their dizziness, whereas nonperipheral patients reported stronger spinning during caloric stimulation. These group differences outperformed those based on the dizziness ratings alone, which likely reflects pronounced caloric symptoms in the nonperipheral group, especially in functional dizziness, rather than stable individual differences. Peripheral patients also reported stronger caloric symptoms than controls without dizziness (*n* = 20). Symptom-specific group differences were not accounted for by slow-phase velocity of caloric nystagmus.

**Conclusions:**

This study highlights the potential diagnostic value of comparing symptoms with caloric stimulation and provides further support for motion perception overestimation in functional dizziness.

**Supplementary Information:**

The online version contains supplementary material available at 10.1007/s00415-025-13334-3.

## Introduction

In clinical practice, the diagnostic assessment of chronic dizziness largely depends on a detailed patient history. In addition to modulating factors such as head movements or complex visual stimuli, and temporal features like the duration of dizziness phases, the symptoms patients experience during these phases are relevant to clinical decision making. These include both the qualitative character of dizziness and associated symptoms such as nausea. Although the diagnostic importance traditionally attributed to symptom quality has declined, the differentiation between spinning and nonspinning motion sensations (vertigo), disturbances of spatial orientation without a sense of motion (dizziness, as defined by the Bárány Society [1]), and unsteadiness still contributes to the diagnostic classification of vestibular disorders [2, 3].

However, attempts to infer the underlying disorder based on the reported quality of dizziness (with "dizziness" used here as an umbrella term) are limited by substantial variability observed both between individuals and within the same individual over time [4]. To address this, we sought to reduce between-subject variability by having each patient compare their subjective experience of dizziness to their experience during the routinely administered caloric test, thereby controlling for stable individual differences in dizziness experience and reporting. The caloric test provides a clearly nonphysiological, standardized peripheral vestibular stimulus and may therefore serve as a proxy for an acute peripheral vestibular disorder. Following this rationale, we tested the hypothesis that patients with a peripheral vestibular disorder would perceive their dizziness as more similar to the caloric stimulation than would patients with dizziness of nonperipheral origin. To address this, we employed newly developed questionnaires specifically designed to assess and compare symptom experiences during dizziness and caloric stimulation.

Yet, we did not find the expected effect: patients with peripheral dizziness did not perceive caloric stimulation as more similar to their typical dizziness than did patients with nonperipheral dizziness. These findings prompted further exploratory analyses, which revealed distinct symptom patterns between groups, with potential diagnostic relevance and implications for pathophysiological models of functional dizziness.

## Methods

### Study population

Patients were prospectively included at the time caloric testing with water was scheduled as part of routine diagnostic work-up (*n* = 143), typically following referral by an ENT or neurology specialist. Testing was performed at varying clinical stages: after an acute dizziness episode, during a chronic phase, or as part of extended evaluation for hearing loss. Of the 143 patients, 16 were excluded due to insufficient caloric response (slow-phase velocity < 10°/s in all four measurements), and 3 were excluded due to incomplete questionnaire data, resulting in a final sample of 124 patients. Among these, 20 did not report dizziness (nondizziness group). All but one of them had hearing impairments; the remaining patient presented with balance complaints without dizziness.

The objective of this study was to compare patients with peripheral and nonperipheral dizziness. Patient classification followed a multistep diagnostic process. Diagnoses were determined by J.K.P. based on medical records and additional dizziness history obtained by S.R., with the Bárány criteria taken into account. Each diagnosis was then assigned to one of four predefined categories: peripheral, central, functional, or nonvestibular. If multiple plausible diagnoses were identified that fell into different nonperipheral categories (i.e., central, functional, or nonvestibular), the patient was assigned to a mixed category. Finally, all patients with central, functional, nonvestibular, or mixed diagnoses were grouped as nonperipheral. Patients who could not be clearly classified as either peripheral or nonperipheral were excluded from further analyses (*n* = 21). Diagnostic distributions of included dizziness patients are presented in Table 1.

**Table 1:** Diagnostic categories and diagnoses of patients with dizziness

	*n*
Peripheral	49
Menière's Disease	26
Acute Unilateral Vestibulopathy	7
Benign Paroxysmal Positional Vertigo	4
Vestibular Paroxysmia	2
Other Peripheral	10

Nonperipheral	34
Central	
Vestibular Migraine	6
Vascular Dizziness	3
Other Central	1
Nonvestibular	
Hemodynamic Orthostatic Dizziness	1
Functional	
Functional Dizziness	14
Central or Functional	5
Nonvestibular or Functional	3
Central or Nonvestibular	1

Based on this categorisation, the peripheral group included 49 patients (24 female, 25 male; 49% female), the nonperipheral group 34 patients (20 female, 14 male; 59% female), with 14 of them classified as having functional dizziness (8 female, 6 male; 57% female), and the nondizziness group included 20 patients (8 female, 12 male; 40% female). Among the 14 patients diagnosed with functional dizziness, 5 fulfilled the diagnostic criteria for persistent postural–perceptual dizziness (PPPD). In the others, the criteria were only partially met, primarily due to a lack of clear evidence for visual dependence.

The mean age was 52.9 years (SD = 13.6) in the peripheral group, 47.1 years (SD = 19.0) in the nonperipheral group, 44.4 years (SD = 16.7) in the functional group, and 53.5 years (SD = 16.7) in the nondizziness group. Compared with the peripheral group, the bootstrapped 95% confidence intervals (CIs) for mean age differences were [− 1.7, 13.0] for the nonperipheral group, [− 0.9, 17.7] for the functional group, and [− 8.7, 7.7] for the nondizziness group. Since all CIs included zero, these results suggest no consistent age differences between groups. Due to data protection regulations, exact ages were not available. Instead, age was recorded in five-year intervals and approximated using the midpoint of each interval (e.g., 28 for the 26–30 interval).

### Caloric testing

Caloric stimulation was performed on both ears using bithermal irrigation (30 °C and 44 °C) with the ATMOS Variotherm (ATMOS MedizinTechnik GmbH & Co. KG, Lenzkirch, Germany). Eye movements were recorded using video-oculography (VO425b Video-Oculography Kit, Interacoustics, Middelfart, Denmark). For each irrigation, the software calculated the average slow-phase velocity (SPV) within a predefined time window representing the most active phase of the nystagmus. These values were then used to compute the mean and maximum SPV across all four stimulations for each patient.

### Questionnaires

The included patients with dizziness were asked to fill out two newly developed questionnaires: (1) a 30-item Dizziness Questionnaire, completed prior to caloric stimulation, assessing subjective experiences during their most intense dizziness episode, and (2) a 30-item Caloric-and-Self-Comparison Questionnaire, completed after caloric stimulation, using the same items, each answered twice—first in reference to symptoms during caloric stimulation, then as a self-comparison rating indicating how the experience differed from their own dizziness. With regard to caloric stimulation, patients were instructed to rate symptom intensity based on their strongest subjective experience—whether it occurred during cold or warm irrigation, and in the left or right ear. Responses for both the dizziness and caloric conditions were given on a six-point scale (0 = not at all, 5 = very severe), with an additional “don’t know” option. For self-comparison, items were rated using the prompt “During my dizziness (episode), I experienced this sensation…” with five response options: “not at all,” “less than during calorics,” “more than during calorics,” “same as during calorics,” and “don’t know.” Patients also completed a global item at the end of the second questionnaire, assessing the overall similarity between the caloric test and their dizziness experience: “Overall, I experienced the caloric test…” rated on a five-point scale from “completely different from my dizziness episode” (5) to “exactly the same as my dizziness episode” (1). Patients without dizziness—typically those with isolated hearing loss—completed a version assessing only experiences during caloric stimulation. The original German versions are available in the Supplementary Information. English translations and item labels used throughout the manuscript are presented in Table 2. The table also includes a categorisation of the items to facilitate interpretation. These categories are based on theoretical and conceptual considerations and could not be empirically validated with the present dataset due to the limited sample size.

**Table 2:**
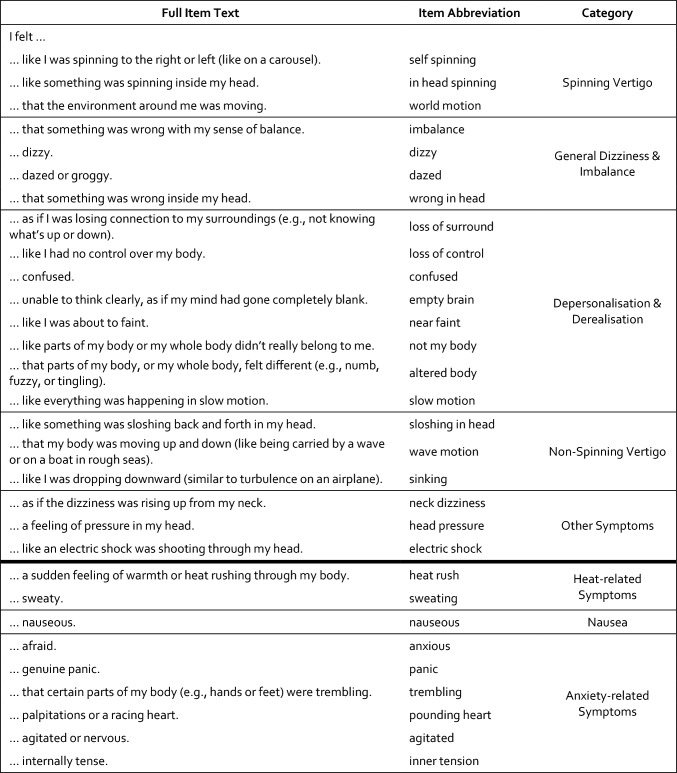
Symptom items

English translation of all symptom items (full text and abbreviation) used to assess experiences during dizziness, caloric stimulation, and self-comparison. For dizziness and caloric stimulation, responses were given on a six-point scale (0 = not at all, 5 = very severe), with a "don't know" option. In the self-comparison, items were rated using the prompt "During my dizziness (episode), I experienced this sensation …" with five response options: "not at all," "less than during calorics," "more than during calorics," "same as during calorics," and "don't know “. Horizontal lines mark boundaries between predefined symptom categories. The thick horizontal line separates two broader sections: 21 items above broadly related to dizziness, and 9 items below reflecting experiences related to heat, nausea, and anxiety

The goal in developing the questionnaires was to comprehensively capture patients’ subjective experiences during a typical dizziness episode using an exploratory approach. The aim was not to construct a scale targeting predefined constructs via a large number of items. Instead, the focus was on a manageable number of items limited to symptoms that could plausibly occur during caloric stimulation. Aspects such as head-movement dependency or symptom frequency were excluded, as they would not apply to the caloric context. To enable meaningful comparison with caloric responses, only symptom intensity was assessed, while temporal aspects were deliberately omitted. At the time of study design (2022), no validated questionnaire met these criteria. The final item set was inspired by the authors’ clinical experience and by established instruments, including the Vertigo Symptom Scale [5], the Cambridge Depersonalisation Scale [6], the State-Trait Anxiety Inventory [7], the Symptom Checklist-90-R [8], the classification of vestibular symptoms by the Bárány Society [1], and by previous work by Sang et al. [9], but all items were newly formulated for the purpose of this study.

### Primary analyses

All primary and exploratory analyses were conducted in MATLAB R2024a using a combination of custom and built-in functions. The primary analyses tested the hypothesis that patients with peripheral dizziness perceive the caloric stimulus as more similar to their own dizziness than patients with nonperipheral dizziness. As part of the study planning, a Bonferroni-corrected significance level of *α* = 0.01 (accounting for five comparisons) and a medium effect size (Cohen’s *d* = 0.6) were assumed. Based on these parameters, a total sample size of 116 participants (58 per group) was calculated to achieve 80% power using G*Power.

In the first analysis, group differences were tested for the global similarity item. For subsequent analyses, the 30 questionnaire items were divided into two sections (see Table 2): a dizziness section comprising 21 items broadly capturing dizziness-related experiences, and an autonomic–anxious section comprising 9 items related to heat, nausea, and anxiety. For each section, two score types were computed: one based on the self-comparison component of the Caloric-and-Self-Comparison Questionnaire, and the other based on the difference between the caloric component and the Dizziness Questionnaire (see below for scoring details). In total, five primary analyses were conducted: one for the global item and four for the two item sections across the two score types. All analyses were evaluated using one-sided unpaired Student’s t tests. Effect sizes were quantified using Cohen’s d, with 95% confidence intervals estimated via nonparametric bootstrapping (10,000 resamples; fixed random seed, rng = 10, to ensure reproducibility).

The Self-Comparison Score was calculated as follows: for each item of the self-comparison component of the Caloric-and-Self-Comparison Questionnaire, a value of 0 was assigned if the symptom was rated as equally strong in both conditions or was not experienced in either condition. A value of 1 was assigned if the symptom was rated as either stronger or weaker during dizziness compared to caloric stimulation, or if it was reported during caloric stimulation but not during dizziness. Items were excluded on a per-patient basis if the response “Don’t know” was selected for the comparison, or if the symptom was absent during dizziness but marked as “Don’t know” for caloric stimulation. For each patient, one Self-Comparison Score was calculated for dizziness symptoms and one for autonomic–anxious symptoms, defined as the mean of all valid item scores within each section. The number of items contributing to the mean varied between patients due to these item-wise exclusions.

The Absolute Difference Score was calculated by computing, for each item, the absolute difference between the ratings on the Dizziness Questionnaire and the caloric component of the Caloric-and-Self-Comparison Questionnaire. Item-level responses were excluded on a per-patient basis if “Don’t know” was selected in either questionnaire. For each patient, the Absolute Difference Score was defined as the mean of all valid item-level differences. As with the Self-Comparison Score, separate scores were computed for dizziness symptoms and autonomic–anxious symptoms.

### Exploratory analyses

Group means and 95% confidence intervals were calculated for each symptom across multiple measures: caloric rating, dizziness rating, difference score (dizziness minus calorics), sum score (dizziness plus calorics), and self-comparison score. Effect sizes were quantified using Cohen’s d and Cohen’s h, and discriminative strength was assessed via the area under the receiver operating characteristic curve (AUC). Confidence intervals for group means, Cohen’s d, Cohen’s h, and AUC values were estimated using nonparametric bootstrapping with 10,000 resamples (fixed random seed: rng = 10, to ensure reproducibility). For group means, Cohen’s d, and Cohen’s h, percentile-based 95% confidence intervals were applied. For AUC values and AUC differences, bias-corrected and accelerated (BCa) 95% confidence intervals were used to account for potential skewness in ROC-based distributions [10].

AUC values were calculated separately for each symptom measure to assess its ability to distinguish between peripheral and nonperipheral (or functional) dizziness. To allow for consistent interpretation of direction, all AUC values were aligned such that values above 0.5 indicated higher scores in the peripheral group, and values below 0.5 indicated higher scores in the nonperipheral (or functional) group.

In a separate analysis, we compared the discriminative strength of two symptom measures by computing the difference between their AUC values. Because this comparison focused solely on which measure performed better, the direction of group separation was not relevant. Therefore, all AUC values were rectified prior to comparison: values below 0.5 were transformed using AUC = 1 − AUC, ensuring that all values reflected discriminative strength irrespective of direction. The rectified AUCs were then used to calculate the difference, and 95% confidence intervals were derived using nonparametric bootstrapping. Confidence intervals that did not include zero suggest a statistically meaningful difference in discriminative performance between the two measures.

Based on these aligned AUC values, we implemented a hierarchical selection procedure to identify the most discriminative symptom measure for each item: First, the difference score was prioritized. It was selected as the best measure for a given symptom if its AUC had a 95% confidence interval that did not span 0.5 (i.e., the entire interval was either ≥ 0.5 or ≤ 0.5), and if its AUC was greater than that of both the caloric and the dizziness ratings. If these conditions were not met, the sum score (dizziness plus caloric ratings) was considered next. It was selected if the 95% confidence interval of its AUC did not span 0.5 and if its AUC was greater than that of both the caloric and dizziness ratings. If neither the difference score nor the sum score met these criteria, the individual AUCs for the caloric and dizziness ratings were evaluated. If the 95% confidence interval of either measure did not span 0.5, the measure with the higher AUC was selected. If both the caloric and dizziness AUCs had 95% confidence intervals that spanned 0.5, the symptom was classified as nonsignificant with respect to group discrimination. The best AUC value for each symptom is reported in Table 3, based on this selection hierarchy. 

## Results

### No greater similarity between self-experienced dizziness and caloric symptoms in peripheral vs. nonperipheral patients

To test the hypothesis that patients with dizziness of peripheral origin perceive caloric stimulation as more similar to their self-experienced dizziness than patients with nonperipheral dizziness, we first analysed responses to a global item. This global item was the final item of the Caloric-and-Self-Comparison Questionnaire, which was completed after caloric stimulation. Lower scores on this item indicated that the caloric test was overall experienced as highly similar to the patient’s self-experienced dizziness. Contrary to our hypothesis, however, patients with peripheral dizziness (*n* = 49) did not perceive the caloric stimulation as more similar to their self-experienced dizziness than patients with nonperipheral dizziness (*n* = 34), t(81) = 1.90, *p* = 0.97. In fact, peripheral patients tended to experience the caloric stimulation as slightly more dissimilar (M ± SD: 4.06 ± 1.07) than nonperipheral patients (3.62 ± 1.02; *d* = 0.42, 95% CI [– 0.01, 0.91]).

A consistent result emerged when comparing scores based on the similarity of individual item responses. Specifically, the Self-Comparison Score calculated for the 21 items broadly capturing dizziness-related experiences was not lower in peripheral patients (M ± SD = 0.54 ± 0.22) compared to nonperipheral patients (0.54 ± 0.19); t(81) = – 0.049, *p* = 0.48, *d* = – 0.01, 95% CI [– 0.43, 0.41]. The same was true for the Absolute Difference Score for dizziness symptoms, which was based on the absolute per-item difference between independent ratings of symptoms during caloric stimulation and self-experienced dizziness, without requiring an explicit self-comparison (M ± SD = 1.59 ± 0.62 vs. 1.37 ± 0.55; t(81) = 1.63, *p* = 0.95, *d* = 0.36, 95% CI [– 0.06, 0.81]). The two scores based on 9 items related to heat, nausea, and anxiety also failed to show the expected group difference: the Self-Comparison Score was not significantly lower in peripheral compared to nonperipheral patients (M ± SD = 0.59 ± 0.26 vs. 0.62 ± 0.20; t(81) = – 0.68, *p* = 0.25, *d* = – 0.15, 95% CI [– 0.57, 0.27]), and the same was true for the Absolute Difference Score (M ± SD = 1.70 ± 0.62 vs. 1.59 ± 0.70; t(81) = 0.76, *p* = 0.77, *d* = 0.17, 95% CI [– 0.27, 0.63]).

In light of this null result, we explored several possibilities: whether an effect might have been missed at the item level; whether the effect might become apparent only after binarizing item responses; whether the negative finding could be explained by differences in caloric excitability, as measured by the slow-phase velocity of caloric nystagmus; or whether the effect would emerge only when comparing peripheral patients with those suffering from functional dizziness—a subgroup of the nonperipheral patients. However, we found no indication that any relevant effect had been overlooked (see Supplementary Information).

### Diagnostic potential of relating self-experienced dizziness symptoms to those induced by caloric testing

**Fig. 1:**
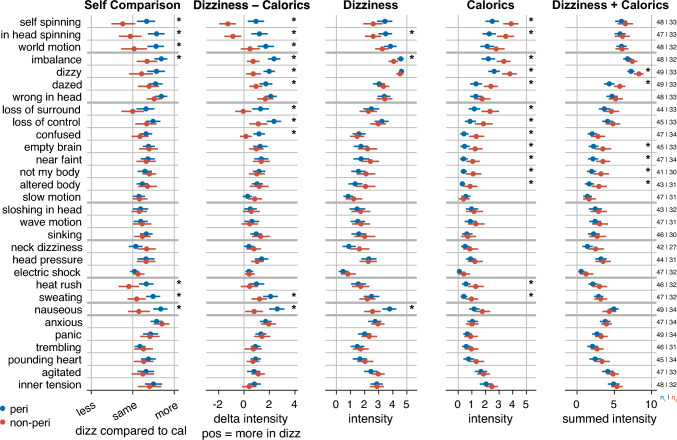
Item-level comparison between peripheral and nonperipheral dizziness groups. For each item, group means and 95% confidence intervals are displayed for five different measures of perceived intensity. The columns differ in how dizziness and calorics are related: (1) direct self-comparison of intensity between dizziness and calorics, (2) calculated difference in intensity between dizziness and calorics, (3) intensity attributed to dizziness, (4) intensity attributed to calorics, and (5) the sum of dizziness and calorics intensity. Items are sorted according to Table 2. Horizontal thick lines mark boundaries between predefined symptom categories. Asterisks indicate that the 95% confidence interval of Cohen’s d for that symptom and measure does not include zero (see Supplementary Information). Sample sizes (n₁: peripheral, n₂: nonperipheral) vary due to “don’t know” responses

As the primary hypothesis could not be confirmed, we used the available data for exploratory analyses aimed at generating hypotheses for future studies. We first asked whether comparing symptom reports across the two conditions—(1) self-experienced dizziness and (2) caloric stimulation—would offer any advantage over the conventional approach of assessing symptoms based solely on self-experienced dizziness. As shown in the second column of Fig. 1, there are symptoms for which patients with peripheral dizziness showed a greater directional difference in symptom intensity (own dizziness minus caloric stimulation) compared to patients with nonperipheral dizziness. In the latter group, the difference was not only smaller but even negative for some symptoms. Group differences were evident for spinning vertigo symptoms, imbalance, nausea, and sweating, as well as for certain symptoms related to general dizziness and depersonalisation/derealisation. In contrast, symptoms related to anxiety and nonspinning vertigo showed no group differences. A similar pattern was observed in the self-comparison component of the Caloric-and-Self-Comparison Questionnaire (“Self Comparison”), as shown in column 1. Notably, these group differences were markedly reduced or even absent when considering only responses referring to the self-experienced dizziness, as presented in the third column of Fig. 1. This exploratory analysis therefore suggests that comparing symptoms between self-experienced dizziness to those during caloric stimulation may indeed offer an advantage and greater diagnostic value than assessing self-experienced dizziness symptoms alone.

What might underlie this apparent advantage? As shown in the fourth column of Fig. 1, patients with nonperipheral dizziness reported many of the assessed symptoms more intensely during caloric stimulation than patients with peripheral dizziness. One might be tempted to conclude that it is the symptom intensity during caloric stimulation, rather than the comparison between conditions, that carries diagnostic value. However, a more differentiated pattern emerges when comparing symptom reports from self-experienced dizziness (column 3) with those elicited by caloric stimulation (column 4). For the three symptoms of spinning vertigo, imbalance, and nausea, a reverse pattern becomes apparent: patients with nonperipheral dizziness reported these symptoms more intensely during caloric stimulation, but less intensely during self-experienced dizziness, as compared to those with peripheral dizziness. This suggests that group differences in both conditions—caloric stimulation and self-experienced dizziness—may amplify one another when analysed using difference scores. In contrast, symptoms related to general dizziness, depersonalisation/derealisation, and heat showed a different pattern: they were also reported more intensely by patients with nonperipheral dizziness during caloric stimulation, but ratings during self-experienced dizziness were comparable to—or even slightly higher than—those of patients with peripheral dizziness. Thus, for these symptoms, it appears that the group differences observed in the comparison derive mainly from caloric stimulation rather than from differences in self-experienced dizziness. For the few symptoms rated more intensely by patients with nonperipheral dizziness in both conditions, the summed intensity across conditions (see column 5) might offer additional diagnostic value.

An ROC-based analysis of discriminative strength, using AUC values, broadly supports the pattern observed in Fig. 1 (see Methods and Supplementary Information). Table 3 provides an overview of which symptom measure—i.e., the difference between dizziness and caloric conditions, dizziness alone, caloric stimulation alone, or their sum—shows the highest discriminative strength for each symptom. Additionally, for each symptom, the higher of the two within-group Spearman correlations between dizziness and caloric ratings is reported as a proxy for stable individual differences. The assumption of such stable individual differences was our primary rationale for conducting the symptom comparison. Indeed, correlation coefficients of 0.4 or higher were observed for many symptoms, which supports the existence of such a stable factor. However, it is also evident that for most symptoms where the difference score provides the highest discriminative strength, both the dizziness and the caloric ratings alone also show group differences. This suggests that, where the symptom comparison outperforms caloric symptoms alone, the advantage may lie more in the presence of a reverse pattern across conditions than in the removal of stable individual differences.

**Table 3:**
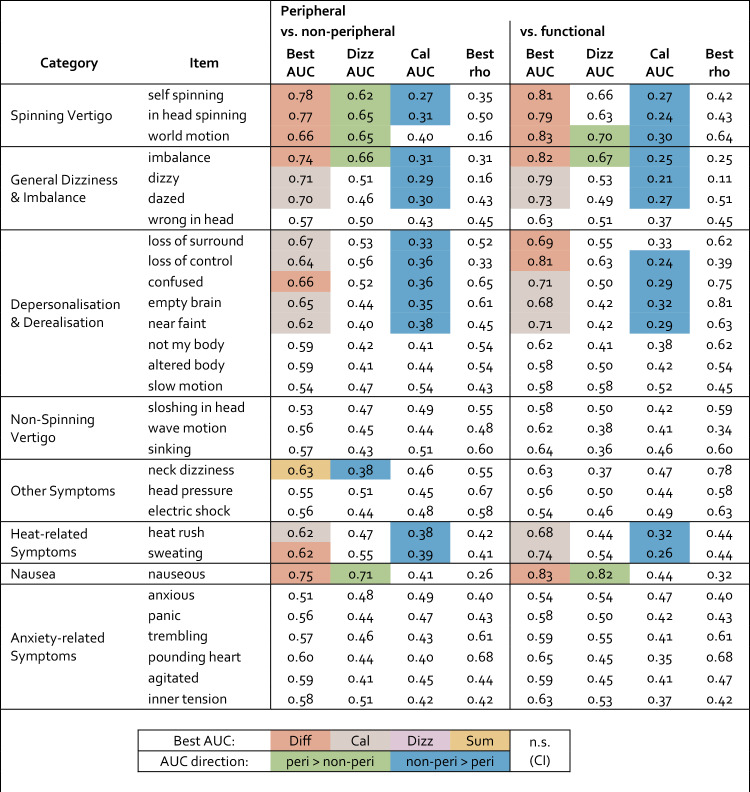
Overview of discriminative strengths and potential contributing factors to combined-measure advantage where present

For each symptom, group discriminability is shown for peripheral vs. nonperipheral (left columns) and peripheral vs. functional dizziness (right columns), along with the strongest correlation between dizziness and caloric ratings. "Dizz AUC" and "Cal AUC" are direction-specific (values > 0.5 indicate higher ratings in the peripheral group), while "Best AUC" represents the most discriminative measure—dizziness, calorics, difference between the two, or their sum (see Methods for details on the selection procedure); directionality is not retained (higher values indicate better discrimination). The color of the Best AUC highlights the selected measure. Green and blue shading in the Dizz AUC or Cal AUC columns marks AUCs whose 95% confidence intervals do not span 0.5. The best correlation refers to the highest Spearman correlation coefficient (rho), calculated separately for each group

The table also includes results from a comparison between peripheral dizziness and functional dizziness patients, a subgroup of the nonperipheral group. This analysis reveals a qualitatively similar pattern. Notably, discriminative strengths are higher for most symptoms, suggesting that the functional subgroup may be primarily driving the observed differences between peripheral and nonperipheral patients (see Supplementary Information for further analyses).

### Beyond diagnostic value: exploring mechanisms behind caloric symptom patterns

Given the apparent diagnostic value of caloric symptoms—especially in distinguishing peripheral from functional dizziness—what underlying mechanisms might explain this finding?

A straightforward physiological explanation would be group differences in caloric excitability, as measured by the slow-phase velocity of caloric nystagmus. However, corresponding analyses showed either no group differences or, where such differences existed, group differences in symptom ratings persisted even after matching for caloric response (see Supplementary Information).

**Fig. 2:**
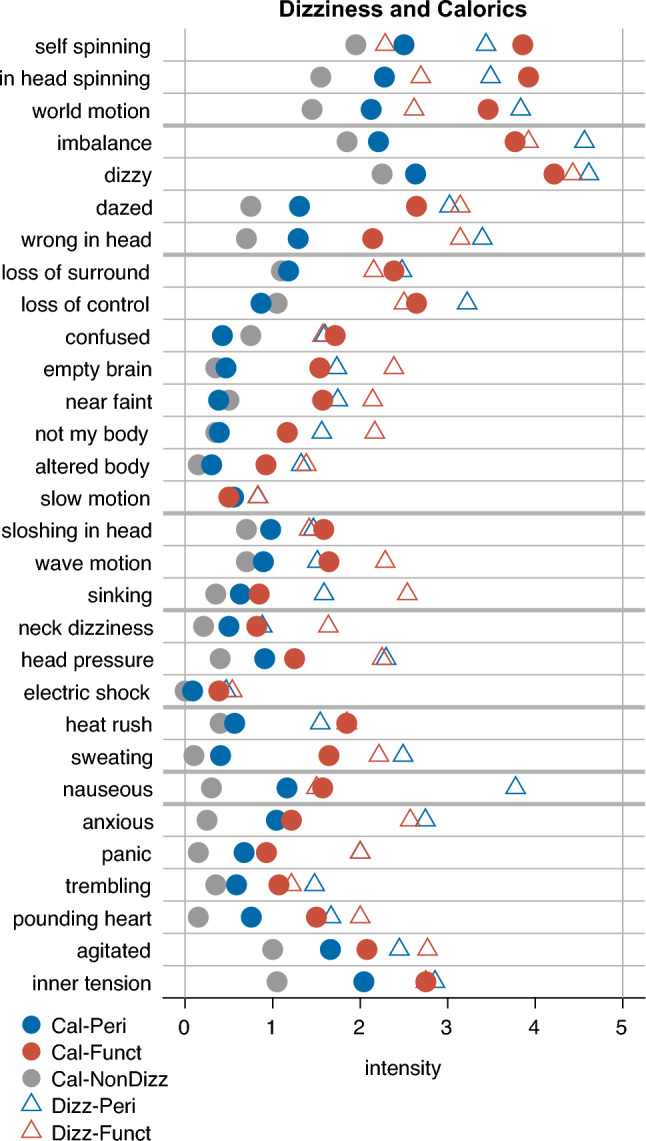
Symptom ratings during dizziness and caloric stimulation relative to a nondizziness reference group. For each item, mean perceived intensities are shown. Colors indicate patient groups (Peri = peripheral, Funct = functional, NonDizz = nondizziness), while symbols differentiate conditions: triangles represent dizziness, circles represent caloric stimulation. Items are grouped by the categories used in Table 3, with thick horizontal lines marking category boundaries for visual separation. The order of categories and the arrangement of items within each category were partially structured to place items with similar caloric response patterns close to each other, improving interpretability

This makes it unlikely that peripheral vestibular responsiveness accounts for the observed symptom differences. Instead, central mechanisms are likely to be involved. To examine the caloric symptom pattern in more detail, Fig. 2 presents a visual comparison of mean caloric symptom ratings from patients with peripheral and functional dizziness, as well as from a nondizzy reference group (*n* = 20); self-experienced dizziness ratings are shown for comparison.

This figure illustrates that patients with functional dizziness reported higher symptom ratings during caloric stimulation across nearly all items, compared to both peripheral dizziness patients and individuals without a history of dizziness. At first glance, this could suggest a general response bias. However, this interpretation appears unlikely, as functional patients rated several symptoms during their own dizziness experience—particularly nausea and spinning vertigo—lower than peripheral patients (see triangles in Fig. 2).

Alternatively, one might assume a general amplification of symptom perception during caloric stimulation. However, this explanation also appears unlikely, as two dissociations argue against it. First, patients with peripheral dizziness reported higher ratings than the nondizzy group for symptoms related to spinning vertigo, general dizziness and imbalance, nausea, and anxiety, but not for heat-related or depersonalisation and derealisation symptoms. Second, while peripheral and functional patients showed only minor differences in nausea and anxiety during caloric stimulation, they differed more clearly in spinning vertigo, general dizziness and imbalance, depersonalisation and derealisation, and heat-related symptoms (see also Table 3). Together, these dissociations suggest a simplified grouping into three symptom clusters: (1) vertigo and dizziness, (2) depersonalisation, derealisation, and heat-related symptoms, and (3) nausea and anxiety. This pattern makes it unlikely that a single amplifying factor—such as general distress or anxiety—fully accounts for the observed symptom profile during caloric stimulation.

Regarding disease specificity, Fig. 2 shows that caloric stimulation elicits stronger vertigo and dizziness symptoms in both patient groups compared to individuals without a history of dizziness. When comparing the increase from the peripheral to the functional group relative to the increase from the nondizzy group to the functional group, clear differences between symptom clusters emerge. While vertigo and dizziness symptoms show a marked relative increase, the rise in nausea and anxiety-related symptoms is comparatively low. In contrast, depersonalisation, derealisation, and heat-related symptoms show a disproportionately high increase. This asymmetry may reflect a characteristic symptom profile in functional dizziness.

## Discussion

The starting point of this study was a well-known diagnostic problem in chronic dizziness: the high interindividual variability in how patients describe the symptoms they experience during dizziness. We investigated whether this variability could be mitigated by comparing the symptoms a patient reports during their dizziness with those experienced during caloric stimulation, under the assumption of stable individual differences in dizziness experience and reporting. Based on the premise that caloric stimulation mimics an acute peripheral vestibular disturbance, this approach was expected to improve the differentiation between peripheral and nonperipheral dizziness. The study was designed accordingly. We hypothesized that the subjective experience during caloric stimulation would closely resemble that of an actual peripheral vestibular disorder. However, the primary analyses comparing the similarity between caloric-induced symptoms and self-experienced dizziness across peripheral and nonperipheral groups revealed effect sizes contrary to our expectations. Instead of the anticipated negative effect size (*d* ≈ – 0.6), the results consistently showed effect sizes close to zero or even slightly positive. Therefore, despite the smaller sample size than originally planned (~ 60 patients per group for 80% power; actual *n* = 49 and *n* = 34 for the peripheral and nonperipheral groups, respectively), and in the absence of any indication of a missed effect in extended analyses, the findings provide sufficient evidence to reject the hypothesis.

While the primary hypothesis—which relied on the absolute difference in symptom intensity as a measure of similarity—could not be confirmed, exploratory analyses suggest that the directed difference between caloric stimulation and self-experienced dizziness may have diagnostic relevance. For the symptoms of spinning vertigo (comprising three items) and imbalance (one item), the diagnostic value of the symptom comparison may stem from a reverse pattern between the groups: patients with peripheral dizziness report higher intensities during self-experienced dizziness, whereas those with nonperipheral dizziness report higher intensities during caloric stimulation. For spinning vertigo, this reverse pattern was so pronounced that it almost suggests a clinical rule of thumb: peripheral patients experience their strongest spinning sensations during their own dizziness, whereas nonperipheral patients—particularly those with functional dizziness—do so during caloric stimulation. It is noteworthy that the reverse pattern seems more critical for the diagnostic value of the symptom comparison than the correction of stable individual differences, despite the latter having been our primary assumption.

Overall, it was unexpected that the contribution of self-experienced dizziness to group differentiation was limited to spinning vertigo symptoms, imbalance, and nausea. Group differentiation based on these symptoms—particularly in comparison to the functional subgroup—is consistent with the diagnostic criteria for Menière’s disease and acute unilateral vestibulopathy on the one hand [3, 11], and PPPD on the other [2], and was therefore to be expected. However, the absence of any contribution from other symptoms—such as those related to nonspinning vertigo or general dizziness—was unexpected and may suggest that these symptoms have limited value in distinguishing peripheral from nonperipheral vestibular disorders.

What stood out most, however, was that group discrimination was strongly driven by differences in caloric symptoms. This effect could not be explained by differences in caloric excitability as measured by slow-phase velocity of caloric nystagmus (SPV). Contrary to our initial assumption that caloric stimulation would be perceived similarly across patient groups and serve as a neutral reference to control for individual differences, it became evident that the diagnostic value of comparing the two conditions largely stems from group differences in symptom intensity during caloric stimulation itself. This was particularly true for symptoms related to general dizziness, depersonalisation/derealisation, and heat, which did not differ between groups with respect to self-experienced dizziness. Such group-specific differences in caloric-induced symptoms have rarely been reported in the absence of SPV differences, except in migraine, where increased dizziness and nausea have been observed [12, 13]. Only recently, a study by Hannigan et al. reported a tendency toward greater dizziness and nausea during caloric stimulation in patients with mal de débarquement syndrome and PPPD—the predominant form of functional dizziness—compared to those with peripheral vestibular disorders [14]. Their study focused on distinguishing vestibular migraine from Menière’s disease. In contrast, we compared peripheral vestibular disorders, including Menière’s disease, with nonperipheral dizziness, including functional dizziness and a small number of vestibular migraine cases. We found particularly pronounced differences between peripheral and functional dizziness. However, due to small subgroup sizes, we could not directly compare Menière’s disease and vestibular migraine. Overall, our findings extend those of Hannigan et al. by showing that caloric symptom patterns may have diagnostic relevance beyond the context of migraine.

In addition, the comparison of symptom patterns across patient groups (peripheral, functional, nondizziness), as illustrated in Fig. 2, provides an indication of what may constitute the specificity of the caloric symptom pattern in functional dizziness. This specificity may lie in the combination of relatively low levels of anxiety-related symptoms and nausea, despite an increased level of vertigo and dizziness symptoms, alongside comparatively strong experiences of depersonalisation, derealisation, and heat-related symptoms. The comparatively low levels of anxiety-related symptoms during caloric stimulation are consistent with current understandings of the pathophysiology of PPPD. While anxiety associated with acute dizziness episodes has been shown to contribute to the development of PPPD, it appears to be less relevant in later stages of the disorder [2, 15, 16]. Regarding the experience of derealisation and depersonalisation, it is well known that such sensations can occur during caloric stimulation [9] and in vestibular disorders [9, 17] including PPPD [18]. Our finding suggests that, given a certain level of vertigo or dizziness, patients with functional dizziness (including PPPD) may be particularly prone to these symptoms.

In principle, elevated ratings of vertigo and dizziness symptoms during caloric stimulation could themselves contribute to the specificity of functional dizziness, supplementing the patterns discussed above. However, since a history of peripheral vestibular disorder alone appears sufficient to elicit stronger perceptions of these symptoms, only the degree of symptom amplification might represent a specific marker. This potential specificity is further limited by reports of elevated ratings in vestibular migraine [14]. Given this limited diagnostic specificity, the finding may itself point to a shared pathophysiological process. It is therefore worth considering whether a common underlying mechanism might be present across all vestibular disorders—potentially modulating the intensity of vertigo and dizziness—and whether this mechanism is simply more pronounced in functional dizziness. Following current models of PPPD pathophysiology, such a mechanism may involve a chronic misperception of motion [15, 19]. Since not all types of motion are misperceived [20–22], this implies a specificity of the underlying mechanism, one that remains poorly understood. Increased motion perception has been observed during galvanic vestibular stimulation, where patients with PPPD perceived body motion at lower current intensities than healthy controls [22, 23], as well as in the perception of swaying [24]. In our study, the three more intense spinning vertigo symptoms reported during caloric stimulation—potentially contributing to the emergence of other symptoms—can be understood as amplified perceptions of rotational motion. In this regard, our findings introduce another condition in which this mechanism appears to be active. A common feature across these conditions is an amplified perception of motion that exceeds both the experiences of healthy individuals in identical test settings and objective behavioral indicators, such as eye movements or postural responses. Irrespective of the exact nature of this overestimation mechanism or how its specificity arises, it is likely to contribute to the potential diagnostic value of caloric symptoms observed in this study—particularly in distinguishing functional dizziness from peripheral vestibular disorders.

Several limitations of the present study should be acknowledged. The study was designed to test the hypothesis that patients with peripheral vestibular disorders would perceive their most severe dizziness experiences as more similar to caloric stimulation than patients with nonperipheral dizziness. Sample size was determined based on a limited number of prespecified primary analyses, which constituted the confirmatory part of the study and clearly rejected the primary hypothesis. All subsequent analyses are exploratory, as they were not prespecified. Many of the observed effects were substantial and consistent. However, given the relatively small sample size, confirmatory analyses are necessary. The provided means and effect sizes with 95% confidence intervals may assist in planning such future studies. Larger samples with a broader representation of different vestibular disorders would allow for more refined and generalizable analyses, including direct comparisons with healthy individuals without a history of inner ear disease or dizziness. Future studies may also re-evaluate the symptom categories used here through factor analysis, which could require an expanded item set. In addition, the potential advantage of self-comparison ratings over separate assessments of dizziness and caloric symptoms warrants further investigation, especially with regard to simple and practical diagnostic applications. Increasing the number of scale points in the self-comparison measure could enhance comparability, while separating it from the caloric symptom ratings may facilitate independent use. Of note, a scenario not observed in our sample—but common in clinical practice and likely to occur with larger samples—is that of patients aborting caloric testing, e.g., due to vomiting. Such cases should be considered separately, as they may carry diagnostic value on their own and were not included in the present analysis.

In summary, the exploratory findings we consider most promising suggest that the assessment of caloric symptoms holds considerable potential—not only for diagnosing vestibular disorders, but also for advancing the pathophysiological understanding of dizziness symptoms, particularly in patients with functional dizziness. For both the development of a simple and practical diagnostic tool and the investigation of underlying mechanisms, caloric stimulation offers a key advantage: it is widely used in clinical routine, and symptom questionnaires can be easily integrated.

## Supplementary Information

Below is the link to the electronic supplementary material.

Supplementary file1 (PDF 2149 KB)

## References

[1] Bisdorff A, Von Brevern M, Lempert T et al (2009) Classification of vestibular symptoms: towards an international classification of vestibular disorders. J Vestib Res 19:1–13. 10.3233/VES-2009-0343 19893191 10.3233/VES-2009-0343

[2] Staab JP, Eckhardt-Henn A, Horii A et al (2017) Diagnostic criteria for persistent postural–perceptual dizziness (PPPD): Consensus document of the committee for the classification of vestibular disorders of the bárány society. J Vestib Res 27:191–208. 10.3233/VES-170622 29036855 10.3233/VES-170622 PMC9249299

[3] Strupp M, Bisdorff A, Furman J et al (2022) Acute unilateral vestibulopathy/vestibular neuritis: diagnostic criteria. J Vestib Res Equilib Orientat 32:389–406. 10.3233/VES-220201 10.3233/VES-220201 PMC9661346 35723133

[4] Newman-Toker DE, Cannon LM, Stofferahn ME et al (2007) Imprecision in patient reports of dizziness symptom quality: a cross-sectional study conducted in an acute care setting. Mayo Clin Proc 82:1329–1340. 10.4065/82.11.1329 17976352 10.4065/82.11.1329

[5] Yardley L, Masson E, Verschuur C et al (1992) Symptoms, anxiety and handicap in dizzy patients: development of the Vertigo symptom scale. J Psychosom Res 36:731–741. 10.1016/0022-3999(92)90131-K 1432863 10.1016/0022-3999(92)90131-k

[6] Sierra M, Berrios GE (2000) The Cambridge Depersonalisation Scale: a new instrument for the measurement of depersonalisation. Psychiatry Res 93:153–164. 10.1016/s0165-1781(00)00100-1 10725532 10.1016/s0165-1781(00)00100-1

[7] Spielberger CD, Gorsuch RL, Lushene RE (1983) Manual for the state-trait anxiety inventory (Form Y). Consulting Psychologists Press, Palo Alto, CA. 10.1037/t06496-000

[8] Franke GH (2011) SCL-90-R: Symptom-Checkliste von L. R. Derogatis – Deutsche Version. Göttingen: Hogrefe

[9] Sang FYP, Jáuregui-Renaud K, Green DA et al (2006) Depersonalisation/derealisation symptoms in vestibular disease. J Neurol Neurosurg Psychiatry 77:760–766. 10.1136/jnnp.2005.075473 16464901 10.1136/jnnp.2005.075473 PMC2077438

[10] Ruscio J, Mullen T (2012) Confidence intervals for the probability of superiority effect size measure and the area under a receiver operating characteristic curve. Multivar Behav Res 47:201–223. 10.1080/00273171.2012.658329 10.1080/00273171.2012.658329 26734848

[11] Lopez-Escamez JA, Carey J, Chung W-H et al (2015) Diagnostic criteria for Menière’s disease. J Vestib Res 25:1–7. 10.3233/VES-150549 25882471 10.3233/VES-150549

[12] Vitkovic J, Paine M, Rance G (2008) Neuro-otological findings in patients with migraine- and nonmigraine-related dizziness. Audiol Neurootol 13:113–122. 10.1159/000111783 18057875 10.1159/000111783

[13] Moran M, Vitkovic J (2013) Perceptual differences in vestibular migraine populations in response to vestibular function testing. Aust N Z J Audiol 33:1–14

[14] Hannigan IP, Rosengren SM, Bharathy GK et al (2024) Subjective and objective responses to caloric stimulation help separate vestibular migraine from other vestibular disorders. J Neurol 271:887–898. 10.1007/s00415-023-12027-z 37847290 10.1007/s00415-023-12027-z PMC10828018

[15] Staab JP (2023) Persistent postural-perceptual dizziness: review and update on key mechanisms of the most common functional neuro-otologic disorder. Neurol Clin 41:647–664. 10.1016/j.ncl.2023.04.003 37775196 10.1016/j.ncl.2023.04.003

[16] Qin C, Zhang R, Yan Z (2025) Research progress on the potential pathogenesis of persistent postural-perceptual dizziness. Brain Behav 15:e70229. 10.1002/brb3.70229 39740787 10.1002/brb3.70229 PMC11688117

[17] Jáuregui-Renaud K, Sang FYP, Gresty MA et al (2008) Depersonalisation/derealisation symptoms and updating orientation in patients with vestibular disease. J Neurol Neurosurg Psychiatry 79:276–283. 10.1136/jnnp.2007.122119 17578858 10.1136/jnnp.2007.122119

[18] Jáuregui-Renaud K, Cabrera-Pereyra R, Miguel-Puga JA et al (2024) Graviception uncertainty, spatial anxiety, and derealization in patients with persistent postural-perceptual dizziness. J Clin Med 13:6665. 10.3390/jcm13226665 39597808 10.3390/jcm13226665 PMC11594595

[19] Arshad Q, Saman Y, Sharif M et al (2021) Magnitude estimates orchestrate hierarchal construction of context-dependent representational maps for vestibular space and time: theoretical implications for functional dizziness. Front Integr Neurosci 15:806940. 10.3389/fnint.2021.806940 35185485 10.3389/fnint.2021.806940 PMC8855482

[20] Pomper JK, Gebert L, Fischer M et al (2013) Does chronic idiopathic dizziness reflect an impairment of sensory predictions of self-motion? Front Neurol 4:181. 10.3389/fneur.2013.00181 24265626 10.3389/fneur.2013.00181 PMC3820974

[21] Kobel MJ, Wagner AR, Oas JG et al (2024) Characterization of vestibular perception in patients with persistent postural-perceptual dizziness. Otol Neurotol Off Publ Am Otol Soc Am Neurotol Soc Eur Acad Otol Neurotol 45:75–82. 10.1097/MAO.0000000000004053 10.1097/MAO.0000000000004053 38013457

[22] Storm R, Krause J, Blüm S-K et al (2024) Visual and vestibular motion perception in persistent postural–perceptual dizziness (PPPD). J Neurol 271:3227–3238. 10.1007/s00415-024-12255-x 38441610 10.1007/s00415-024-12255-x PMC11136745

[23] Woll J, Sprenger A, Helmchen C (2019) Postural control during galvanic vestibular stimulation in patients with persistent perceptual–postural dizziness. J Neurol 266:1236–1249. 10.1007/s00415-019-09255-7 30809703 10.1007/s00415-019-09255-7

[24] San Pedro Murillo E, Bancroft MJ, Koohi N et al (2023) Postural misperception: a biomarker for persistent postural perceptual dizziness. J Neurol Neurosurg Psychiatry 94:165–166. 10.1136/jnnp-2022-329321 35995549 10.1136/jnnp-2022-329321

