# Supplementary material for: Comparing caloric and self-experienced dizziness symptoms: diagnostic value and implications in functional dizziness
Source: J Neurol. 2025 Aug 30;272(9):600. doi: 10.1007/s00415-025-13334-3 (PMC12398442; doi:10.1007/s00415-025-13334-3)
Supplement: Supplementary file 1 — Supplementary file1 (PDF 2149 KB) [file 415_2025_13334_MOESM1_ESM.pdf]

Supplementary Information

The following Supplementary Information serve three main purposes: (1) to offer a detailed account of the analyses underlying the results reported in the main text, (2) to present effect size estimates (Cohen's d, Cohen's h, and AUC values) along with their 95% confidence intervals to support the planning of confirmatory studies, and (3) to provide the original German versions of the newly developed questionnaires.

The information is organised as follows:

1) Exploratory analyses supporting the conclusion that the hypothesis of greater similarity between caloric symptoms and self-experienced dizziness in peripheral patients – compared to non-peripheral (and functional) patients – should be rejected.

2) Exploratory analyses assessing the diagnostic value of comparing symptoms during self-experienced dizziness with those induced by caloric stimulation.

3) Analyses demonstrating that group differences in the experience of caloric stimulation cannot be explained by differences in the slow-phase velocity of caloric nystagmus.

4) Original German versions of the Dizziness Questionnaire and the Caloric-and-Self-Comparison Questionnaire.

1) Peripheral patients do not perceive caloric stimulation as more similar to their symptoms than non-peripheral or functional patients

1a) Symptom-specific similarity: self-comparison

In an exploratory item-level analysis, we computed Cohen's h (with 95% confidence intervals) for each symptom to assess whether some symptoms were rated as more similar in the peripheral group compared to the non-peripheral group (see Supplementary Table 1, Self-Comparison column). For most items ($n = 18$), $|h|$ was below 0.25 and confidence intervals included zero. Among the remaining 12 items with $|h| > 0.25$, effects varied in direction and most confidence intervals included zero. Thus, no consistent pattern emerged indicating greater perceived similarity in the peripheral group.

1b) Symptom-specific similarity: absolute difference

An exploratory item-level analysis, analogous to the self-comparison analysis, also showed no clear indication of greater perceived similarity in the peripheral group (see Supplementary Table 1, ordinal column under |Dizziness – Calorics|). Instead, among Cohen's d values with $|d| \geq 0.25$, more were positive than negative, suggesting a trend toward greater perceived similarity in the non-peripheral group.

1c) Symptom presence vs. absence: binarized similarity scores

We further explored whether perceived similarity might be better captured qualitatively – by assessing whether a symptom was present or absent. To this end, we binarized the Likert-scale ratings from the caloric and dizziness questionnaires using two thresholds:

(1) 0 = absent; 1–5 = present

(2) 0–1 = absent; 2–5 = present

Using these binarized scores, we recalculated Absolute Difference Scores. For dizziness symptoms, no indication of the expected group effect emerged:

– Approach 1: M ± SD = 0.32 ± 0.17 (peripheral) vs. 0.26 ± 0.17 (non-peripheral); d = 0.34, 95% CI [–0.11, 0.81]

– Approach 2: 0.37 ± 0.18 vs. 0.28 ± 0.18; d = 0.53, 95% CI [0.09, 1.00]

Similarly, no relevant group differences were observed for autonomic-anxious symptoms:

– Approach 1: 0.38 ± 0.22 vs. 0.35 ± 0.23; d = 0.17, 95% CI [–0.27, 0.62]

– Approach 2: 0.43 ± 0.20 vs. 0.39 ± 0.24; d = 0.16, 95% CI [–0.28, 0.63]

An exploratory item-level analysis based on both binarization approaches (see Supplementary Table 1, binary columns) also revealed no consistent indication of greater similarity in the peripheral group. In fact, most Cohen's h values with $|h| \geq 0.25$ were positive, suggesting greater perceived similarity in the non-peripheral group.

Supplementary Table 1. Symptom dissimilarity between dizziness and caloric stimulation: item-level comparison between peripheral and non-peripheral dizziness groups. For each item, effect sizes (peripheral – non-peripheral) and 95% confidence intervals (CIs) are shown. Depending on the scale of the difference variable, either Cohen's d (ordinal) or Cohen's h (binary) was used. In the Self-Comparison column, binary values indicate whether a difference between dizziness and caloric experience was reported (1 = different, 0 = same). For |Dizziness – Calorics|, the ordinal version reflects the absolute difference between two Likert-scale ratings (0–5), yielding values from 0 to 5. The binary version was based on presence/absence coding of both ratings prior to comparison, using either Approach 1 (0 → 0, 1–5 → 1) or Approach 2 (0–1 → 0, 2–5 → 1). Positive values indicate greater dissimilarity in the peripheral group, negative values in the non-peripheral group. Items with |d| or |h| > 0.25 are highlighted (green = peripheral > non-peripheral; blue = non-peripheral > peripheral, as hypothesized). Bold indicates that the 95% CI excluded zero in at least one comparison. Sample sizes (n_1: peripheral, n_2: non-peripheral) vary due to "don't know" responses. Horizontal lines mark boundaries between predefined symptom categories. The thick horizontal line separates dizziness from autonomic-anxious symptoms. Items are sorted according to Table 2.

Item	Self-Comparison Binary Cohen's h	n_1	n_2	|Dizziness – Calorics| Ordinal Cohen's d	Binary (App. 1) Cohen's h	Binary (App. 2) Cohen's h	n_1	n_2
self spinning	-0.09 [-0.56, 0.36]	48	33	0.14 [-0.31, 0.59]	0.01 [-0.43, 0.49]	-0.03 [-0.49, 0.44]	48	34
in head spinning	0.36 [-0.09, 0.82]	48	33	0.18 [-0.26, 0.61]	-0.13 [-0.56, 0.32]	0.22 [-0.23, 0.67]	48	34
world motion	0.12 [-0.34, 0.55]	48	34	0.40 [-0.03, 0.85]	0.34 [-0.11, 0.81]	0.38 [-0.04, 0.85]	48	32
**imbalance**	0.37 [-0.09, 0.80]	49	32	0.72 [0.31, 1.19]	0.35 [-0.08, 0.84]	0.53 [0.11, 1.06]	48	34
**dizzy**	0.38 [-0.08, 0.86]	49	33	0.64 [0.20, 1.18]	-0.12 [-0.60, 0.37]	0.43 [-0.00, 0.94]	49	34
**dazed**	-0.27 [-0.73, 0.17]	49	33	0.30 [-0.11, 0.74]	0.60 [0.18, 1.08]	0.36 [-0.07, 0.81]	49	34
wrong in head	0.32 [-0.11, 0.77]	48	33	0.20 [-0.24, 0.66]	0.13 [-0.31, 0.60]	0.32 [-0.10, 0.77]	49	34
**loss of surround**	0.07 [-0.38, 0.52]	46	34	0.39 [-0.02, 0.83]	0.80 [0.36, 1.33]	0.54 [0.10, 1.01]	47	33
**loss of control**	-0.14 [-0.60, 0.30]	47	34	0.40 [-0.03, 0.87]	0.53 [0.09, 1.00]	0.43 [-0.01, 0.90]	47	33
**confused**	-0.02 [-0.45, 0.43]	47	34	0.24 [-0.17, 0.64]	0.46 [0.03, 0.93]	0.44 [0.00, 0.91]	49	34
**empty brain**	-0.42 [-0.87, 0.01]	46	33	0.20 [-0.22, 0.64]	0.45 [0.01, 0.92]	0.37 [-0.08, 0.84]	47	33
near faint	-0.28 [-0.73, 0.17]	48	34	-0.10 [-0.54, 0.34]	-0.06 [-0.50, 0.39]	-0.03 [-0.48, 0.42]	48	34
not my body	-0.03 [-0.49, 0.43]	45	33	-0.02 [-0.49, 0.42]	0.18 [-0.28, 0.66]	0.40 [-0.06, 0.89]	44	31
**altered body**	-0.49 [-0.94, -0.04]	48	32	-0.30 [-0.79, 0.15]	-0.13 [-0.60, 0.34]	-0.02 [-0.48, 0.45]	44	33
slow motion	0.01 [-0.44, 0.48]	47	32	-0.16 [-0.62, 0.30]	-0.10 [-0.56, 0.36]	-0.13 [-0.57, 0.33]	49	32
sloshing in head	0.13 [-0.32, 0.58]	47	32	-0.04 [-0.50, 0.42]	0.00 [-0.44, 0.48]	0.00 [-0.45, 0.48]	44	32
wave motion	-0.16 [-0.62, 0.28]	47	33	0.15 [-0.29, 0.61]	0.02 [-0.43, 0.49]	0.21 [-0.23, 0.67]	48	31
sinking	0.13 [-0.33, 0.62]	48	31	-0.19 [-0.66, 0.30]	-0.37 [-0.83, 0.09]	0.11 [-0.36, 0.56]	47	31
neck dizziness	-0.01 [-0.49, 0.45]	43	31	-0.39 [-0.96, 0.09]	-0.41 [-0.89, 0.07]	-0.28 [-0.75, 0.21]	45	28
head pressure	0.06 [-0.38, 0.52]	47	32	0.32 [-0.11, 0.76]	0.18 [-0.28, 0.66]	0.05 [-0.40, 0.52]	45	32
electric shock	-0.15 [-0.61, 0.31]	47	33	-0.04 [-0.50, 0.39]	-0.18 [-0.64, 0.28]	-0.06 [-0.52, 0.44]	49	33
heat rush	-0.15 [-0.61, 0.32]	46	32	0.16 [-0.27, 0.59]	0.26 [-0.18, 0.70]	0.13 [-0.31, 0.58]	49	34
sweating	0.27 [-0.17, 0.73]	47	33	0.42 [-0.01, 0.90]	0.14 [-0.31, 0.58]	0.24 [-0.21, 0.71]	47	33
**nauseous**	0.50 [0.06, 0.95]	49	34	0.83 [0.39, 1.35]	0.30 [-0.13, 0.76]	0.48 [0.05, 0.93]	49	34
**anxious**	-0.49 [-0.97, -0.06]	48	34	-0.26 [-0.70, 0.16]	-0.11 [-0.56, 0.34]	-0.32 [-0.79, 0.11]	48	34
panic	-0.11 [-0.57, 0.32]	49	34	-0.25 [-0.71, 0.18]	-0.24 [-0.68, 0.20]	-0.08 [-0.52, 0.36]	49	34
trembling	-0.36 [-0.81, 0.08]	47	33	-0.23 [-0.72, 0.24]	-0.08 [-0.54, 0.39]	-0.24 [-0.69, 0.24]	47	31
pounding heart	-0.18 [-0.64, 0.26]	45	34	-0.06 [-0.51, 0.38]	0.03 [-0.42, 0.49]	0.13 [-0.31, 0.62]	48	34
agitated	-0.13 [-0.60, 0.32]	48	33	-0.03 [-0.47, 0.38]	0.19 [-0.25, 0.63]	0.11 [-0.35, 0.55]	47	34
inner tension	-0.14 [-0.64, 0.32]	48	32	0.04 [-0.40, 0.45]	0.29 [-0.14, 0.78]	0.22 [-0.21, 0.69]	49	34

1d) Controlling for caloric excitability: subgroup analysis based on slow-phase velocity

Our primary hypothesis rested on the assumption that caloric stimulation provides an equal and standardized vestibular input across both patient groups – serving as an identical simulation of a peripheral vestibular disturbance that may be perceived and/or reported differently depending on individual subjective factors. Since our hypothesis was not supported by the data, the underlying assumption may be incorrect. In patients with peripheral dizziness, caloric stimulation may evoke a weaker vestibular response, making it feel qualitatively different or less intense than their typical dizziness.

Indeed, when comparing the mean slow-phase velocity (SPV) of caloric nystagmus across all four stimulations (cold/warm water, left/right ear), the peripheral group showed lower values (M = 13.0 °/s, SD = 6.6) than the non-peripheral group (M = 16.1 °/s, SD = 6.1; Cohen's d = –0.48, 95% CI [–0.98, –0.06]). Notably, this difference was not observed for the maximum SPV across the same stimulations: 21.9 °/s (SD = 11.3) in the peripheral group vs. 22.5 °/s (SD = 8.3) in the non-peripheral group (Cohen's d = –0.06, 95% CI [–0.54, 0.33]).

To examine whether inadequate caloric excitability might have masked a group difference, we included only those patients from both groups whose mean SPV exceeded a predefined threshold, set at the median of the original non-peripheral group (14.25 °/s). In these matched subgroups (n_1 = 17, n_2 = 18), mean SPV no longer differed between groups: the peripheral group showed a mean of 20.7 °/s (SD = 4.8), and the non-peripheral group 20.9 °/s (SD = 4.2; Cohen's d = –0.05, 95% CI [–0.75, 0.63]). We then compared subjective symptom ratings between the two subgroups. However, we found no relevant group differences in the global item (M ± SD: 4.06 ± 1.09 vs. 3.61 ± 1.04; Cohen's d = 0.42, 95% CI [–0.21, 1.25]), in the Self-Comparison Score for dizziness symptoms (M ± SD: 0.50 ± 0.23 vs. 0.55 ± 0.16; Cohen's d = –0.24, 95% CI [–1.01, 0.43]) and for autonomic-anxious symptoms (M ± SD: 0.56 ± 0.19 vs. 0.61 ± 0.21; Cohen's d = –0.26, 95% CI [–1.00, 0.38]), or in the Absolute Difference Score for dizziness symptoms (M ± SD: 1.50 ± 0.65 vs. 1.41 ± 0.45; Cohen's d = 0.15, 95% CI [–0.57, 0.81]) and for autonomic-anxious symptoms (M ± SD: 1.58 ± 0.67 vs. 1.46 ± 0.76; Cohen's d = 0.18, 95% CI [–0.48, 0.88]). Hence, the small difference in mean slow-phase velocity does not explain the absence of the expected group effect.

1e) Subgroup comparison: peripheral vs. functional dizziness

A potential concern is that symptom differences between peripheral and non-peripheral dizziness may be primarily driven by patients with functional dizziness, a distinct subgroup within the non-peripheral category. To explore this, we repeated most of the previous analyses using only the functional subgroup (n = 14). Again, there was no indication that patients with peripheral dizziness perceived caloric stimulation as more similar to their own dizziness than did patients with functional dizziness. If anything, the pattern was more pronounced in the opposite direction: patients with functional dizziness tended to report greater similarity between caloric stimulation and their typical dizziness (see Supplementary Tables 2 and 3).

Supplementary Table 2. Symptom dissimilarity between dizziness and caloric stimulation: score- and global-level comparison between peripheral and functional dizziness groups. For the global item and for scores calculated for dizziness symptoms and autonomic-anxious symptoms, Cohen's d effect sizes (peripheral – functional) and 95% confidence intervals (CIs) are shown. In the Self-Comparison column, binary values indicate whether a difference between dizziness and caloric experience was reported (1 = different, 0 = same). For |Dizziness – Calorics|, the ordinal version reflects the absolute difference between two Likert-scale ratings (0–5), resulting in values from 0 to 5. The binary versions were based on presence/absence coding of both ratings prior to comparison, using either Approach 1 (0 → 0, 1–5 → 1) or Approach 2 (0–1 → 0, 2–5 → 1). Positive values indicate greater dissimilarity in the peripheral group; negative values indicate greater dissimilarity in the functional group. Values with |d| > 0.25 are highlighted: green indicates greater dissimilarity in the peripheral group compared to the functional group (contrary to the hypothesis). Bolded values indicate that the 95% CI excluded zero. Sample sizes: n_1 = peripheral group, n_2 = functional dizziness subgroup.

Measure	Self-Comparison			|Dizziness – Calorics|				
	Binary<br>Cohen's d	n_1	n_2	Ordinal<br>Cohen's d	Binary (App. 1)<br>Cohen's d	Binary (App. 2)<br>Cohen's d	n_1	n_2
global item	0.47 [-0.08 1.14]	49	14	-	-	-	-	-
dizziness symptoms	0.26 [-0.23 0.77]	49	14	0.49 [-0.01 1.01]	**0.50 [0.08 0.93]**	**0.93 [0.47 1.45]**	49	14
autonomic-anxious symptoms	-0.07 [-0.60 0.48]	49	14	0.37 [-0.22 0.98]	0.31 [-0.19 0.84]	0.51 [-0.03 1.08]	49	14

Supplementary Table 3. Symptom dissimilarity between dizziness and caloric stimulation: item-level comparison between peripheral and functional dizziness groups. Layout and interpretation follow that of Supplementary Table 1. The non-peripheral group was replaced by the subgroup of patients diagnosed with functional dizziness.

Item	Self-Comparison Binary Cohen's h	n_1	n_2	|Dizziness – Calorics| Ordinal Cohen's d	Binary (App. 1) Cohen's h	Binary (App. 2) Cohen's h	n_1	n_2
self spinning	-0.08 [-0.95, 0.49]	48	14	-0.03 [-0.66, 0.57]	-0.01 [-0.59, 0.78]	-0.23 [-0.86, 0.41]	48	14
in head spinning	0.50 [-0.14, 1.12]	48	13	-0.01 [-0.60, 0.55]	-0.18 [-0.79, 0.46]	0.21 [-0.40, 0.86]	48	14
**world motion**	0.07 [-0.78, 0.64]	48	14	0.70 [0.25, 1.21]	0.42 [-0.15, 1.32]	0.76 [0.18, 1.70]	48	13
**imbalance**	0.61 [-0.03, 1.25]	49	13	0.70 [0.26, 1.18]	0.27 [-0.29, 1.14]	0.69 [0.13, 1.45]	48	14
**dizzy**	0.31 [-0.58, 0.93]	49	14	0.63 [0.02, 1.27]	0.04 [-0.55, 0.78]	0.59 [0.06, 1.34]	49	14
**dazed**	-0.02 [-0.67, 0.58]	49	14	0.42 [0.02, 0.84]	0.84 [0.31, 1.59]	0.97 [0.42, 1.71]	49	14
**wrong in head**	0.62 [0.01, 1.27]	48	14	0.33 [-0.29, 0.99]	0.38 [-0.19, 1.26]	0.43 [-0.16, 1.09]	49	14
loss of surround	0.02 [-0.63, 0.64]	46	14	0.33 [-0.18, 0.85]	0.50 [-0.12, 1.38]	0.30 [-0.34, 0.96]	47	13
**loss of control**	0.14 [-0.50, 0.75]	47	14	0.61 [0.12, 1.18]	0.99 [0.42, 1.90]	0.89 [0.34, 1.76]	47	14
**confused**	0.10 [-0.51, 0.75]	47	14	0.32 [-0.15, 0.74]	0.51 [-0.08, 1.43]	0.76 [0.21, 1.51]	49	14
**empty brain**	-0.19 [-0.83, 0.45]	46	13	0.31 [-0.18, 0.77]	0.55 [-0.06, 1.46]	0.90 [0.33, 1.68]	47	13
near faint	-0.19 [-0.82, 0.42]	48	14	0.19 [-0.30, 0.67]	0.31 [-0.25, 1.19]	0.23 [-0.38, 0.90]	48	14
not my body	0.04 [-0.58, 0.68]	45	14	-0.01 [-0.62, 0.54]	0.20 [-0.42, 1.10]	0.55 [-0.06, 1.48]	44	12
altered body	-0.14 [-0.72, 0.50]	48	14	-0.05 [-0.76, 0.57]	-0.08 [-0.70, 0.59]	0.15 [-0.46, 1.03]	44	13
slow motion	0.36 [-0.25, 1.33]	47	12	0.14 [-0.54, 0.77]	0.28 [-0.32, 1.17]	0.32 [-0.28, 1.22]	49	13
sloshing in head	0.15 [-0.49, 0.88]	47	12	0.05 [-0.63, 0.66]	0.21 [-0.41, 1.15]	0.21 [-0.41, 1.15]	44	12
wave motion	-0.19 [-0.80, 0.42]	47	14	-0.11 [-0.78, 0.51]	-0.20 [-0.81, 0.44]	0.15 [-0.43, 0.82]	48	14
sinking	-0.02 [-0.63, 0.61]	48	14	-0.54 [-1.34, 0.15]	-0.54 [-1.18, 0.11]	-0.09 [-0.70, 0.55]	47	13
neck dizziness	0.03 [-0.62, 0.73]	43	12	-0.16 [-0.92, 0.38]	-0.23 [-0.87, 0.75]	0.05 [-0.61, 1.03]	45	11
head pressure	0.46 [-0.14, 1.16]	47	13	0.32 [-0.15, 0.79]	-0.12 [-0.77, 0.52]	0.03 [-0.60, 0.70]	45	13
electric shock	0.17 [-0.41, 0.91]	47	13	0.17 [-0.34, 0.50]	-0.19 [-0.78, 0.71]	0.11 [-0.46, 0.83]	49	14
heat rush	-0.16 [-0.80, 0.48]	46	13	0.14 [-0.38, 0.63]	0.34 [-0.23, 1.01]	0.38 [-0.20, 1.22]	49	14
**sweating**	0.24 [-0.38, 0.86]	47	14	0.46 [-0.07, 1.02]	0.40 [-0.21, 1.05]	0.76 [0.19, 1.63]	47	14
**nauseous**	0.60 [-0.02, 1.21]	49	14	0.95 [0.44, 1.56]	0.23 [-0.37, 0.86]	0.63 [0.04, 1.30]	49	14
anxious	-0.23 [-0.88, 0.35]	48	14	0.11 [-0.45, 0.67]	-0.17 [-0.78, 0.44]	0.19 [-0.40, 0.82]	48	14
panic	-0.11 [-0.76, 0.49]	49	14	-0.18 [-0.81, 0.40]	-0.20 [-0.83, 0.39]	0.00 [-0.60, 0.61]	49	14
trembling	-0.22 [-0.84, 0.43]	47	14	-0.20 [-0.92, 0.54]	0.07 [-0.51, 0.75]	-0.17 [-0.77, 0.47]	47	14
pounding heart	-0.36 [-1.01, 0.25]	45	14	-0.13 [-0.78, 0.47]	0.08 [-0.50, 0.95]	0.18 [-0.41, 1.05]	48	14
agitated	-0.18 [-0.88, 0.44]	48	13	0.11 [-0.40, 0.58]	0.24 [-0.34, 1.11]	-0.05 [-0.66, 0.59]	47	14
inner tension	-0.11 [-1.05, 0.51]	48	12	0.06 [-0.49, 0.57]	0.45 [-0.13, 1.17]	0.02 [-0.59, 0.65]	49	14

2) Diagnostic value of comparing symptoms during self-experienced dizziness and caloric stimulation

2a) Effect sizes (Cohen's d and AUC values) from item-level comparisons between peripheral and non-peripheral patient groups

The following two tables present effect sizes (Cohen's d in Supplementary Table 4 and AUC values in Supplementary Table 5) for each symptom measure, following the column structure of Figure 1. In contrast to the similarity analysis, the directional (not absolute) difference between dizziness and calorics was used for the group comparison (column 2).

Supplementary Table 4. Cohen's d from item-level comparisons between peripheral and non-peripheral dizziness groups. For each item and each measure of perceived intensity (see Figure 1), Cohen's d along with the corresponding 95% confidence interval is reported. Item labels in bold indicate that the 95% confidence interval of Cohen's d does not include zero for at least one measure. Cells with color shading indicate effect sizes with an absolute value greater than or equal to 0.25; green denotes higher values in the peripheral dizziness group, whereas blue denotes higher values in the non-peripheral dizziness group. Items are sorted according to Table 2.

Item	Self-Comparison	Dizziness – Calorics	Dizziness	Calorics	Dizziness + Calorics	n_1	n_2
**self spinning**	0.69 [0.23, 1.24]	1.04 [0.61, 1.55]	0.43 [-0.01, 0.92]	-0.80 [-1.35, -0.33]	-0.18 [-0.65, 0.27]	48	33
**in head spinning**	0.87 [0.39, 1.47]	0.98 [0.54, 1.49]	0.51 [0.06, 1.03]	-0.67 [-1.23, -0.20]	-0.11 [-0.59, 0.36]	47	33
**world motion**	0.67 [0.21, 1.23]	0.55 [0.11, 1.06]	0.38 [-0.05, 0.90]	-0.36 [-0.85, 0.09]	-0.03 [-0.50, 0.43]	48	32
**imbalance**	0.52 [0.06, 1.05]	0.98 [0.54, 1.48]	0.56 [0.13, 1.09]	-0.68 [-1.20, -0.23]	-0.30 [-0.81, 0.15]	48	32
**dizzy**	0.45 [-0.01, 0.96]	0.75 [0.29, 1.32]	0.14 [-0.33, 0.59]	-0.73 [-1.29, -0.27]	-0.59 [-1.11, -0.14]	49	33
**dazed**	0.23 [-0.24, 0.70]	0.46 [0.03, 0.90]	-0.22 [-0.64, 0.20]	-0.71 [-1.25, -0.27]	-0.58 [-1.06, -0.15]	49	33
wrong in head	0.32 [-0.13, 0.82]	0.25 [-0.21, 0.71]	-0.02 [-0.47, 0.43]	-0.28 [-0.77, 0.16]	-0.18 [-0.65, 0.27]	48	33
**loss of surround**	0.42 [-0.03, 0.93]	0.65 [0.22, 1.14]	0.11 [-0.35, 0.56]	-0.69 [-1.24, -0.22]	-0.33 [-0.84, 0.13]	44	33
**loss of control**	0.22 [-0.23, 0.69]	0.60 [0.15, 1.09]	0.15 [-0.30, 0.62]	-0.58 [-1.10, -0.13]	-0.27 [-0.76, 0.19]	45	33
**confused**	0.24 [-0.21, 0.71]	0.67 [0.25, 1.10]	0.08 [-0.36, 0.52]	-0.65 [-1.19, -0.18]	-0.31 [-0.80, 0.15]	47	34
**empty brain**	-0.03 [-0.54, 0.44]	0.16 [-0.28, 0.62]	-0.25 [-0.76, 0.20]	-0.57 [-1.12, -0.12]	-0.46 [-0.96, -0.00]	45	33
**near faint**	0.06 [-0.41, 0.52]	0.00 [-0.44, 0.45]	-0.35 [-0.83, 0.09]	-0.53 [-1.04, -0.08]	-0.51 [-1.01, -0.07]	47	34
**not my body**	-0.20 [-0.67, 0.27]	0.10 [-0.37, 0.59]	-0.30 [-0.82, 0.16]	-0.59 [-1.06, -0.10]	-0.50 [-1.03, -0.01]	41	30
**altered body**	-0.22 [-0.78, 0.27]	-0.10 [-0.62, 0.38]	-0.40 [-0.94, 0.06]	-0.51 [-0.95, -0.03]	-0.53 [-1.06, -0.06]	43	31
slow motion	-0.02 [-0.48, 0.42]	-0.39 [-0.85, 0.07]	-0.27 [-0.76, 0.21]	0.14 [-0.31, 0.62]	-0.10 [-0.58, 0.38]	47	31
sloshing in head	0.05 [-0.40, 0.52]	-0.04 [-0.53, 0.42]	-0.13 [-0.63, 0.33]	-0.11 [-0.59, 0.38]	-0.14 [-0.63, 0.33]	43	32
wave motion	-0.05 [-0.53, 0.41]	0.08 [-0.36, 0.56]	-0.12 [-0.60, 0.33]	-0.24 [-0.73, 0.22]	-0.22 [-0.69, 0.24]	47	31
sinking	0.16 [-0.29, 0.65]	-0.19 [-0.67, 0.30]	-0.21 [-0.70, 0.26]	-0.05 [-0.52, 0.45]	-0.17 [-0.65, 0.31]	46	30
neck dizziness	-0.45 [-0.95, 0.03]	-0.28 [-0.89, 0.20]	-0.44 [-1.01, 0.05]	-0.29 [-0.80, 0.21]	-0.43 [-0.95, 0.07]	42	27
head pressure	-0.01 [-0.47, 0.46]	0.22 [-0.22, 0.66]	0.02 [-0.44, 0.49]	-0.23 [-0.72, 0.24]	-0.10 [-0.58, 0.38]	44	31
electric shock	-0.21 [-0.69, 0.25]	-0.02 [-0.49, 0.43]	-0.25 [-0.73, 0.21]	-0.37 [-0.72, 0.27]	-0.33 [-0.75, 0.16]	47	32
**heat rush**	0.64 [0.18, 1.16]	0.26 [-0.18, 0.73]	-0.10 [-0.57, 0.35]	-0.53 [-1.02, -0.06]	-0.36 [-0.84, 0.12]	46	32
**sweating**	0.61 [0.16, 1.14]	0.44 [0.01, 0.92]	0.16 [-0.28, 0.62]	-0.49 [-0.98, -0.04]	-0.11 [-0.57, 0.36]	47	32
**nauseous**	0.76 [0.30, 1.33]	0.84 [0.39, 1.44]	0.70 [0.25, 1.26]	-0.38 [-0.88, 0.06]	0.24 [-0.21, 0.74]	49	34
anxious	-0.24 [-0.75, 0.20]	-0.14 [-0.62, 0.30]	-0.13 [-0.57, 0.31]	0.03 [-0.40, 0.49]	-0.06 [-0.50, 0.37]	47	34
panic	0.03 [-0.42, 0.48]	-0.05 [-0.51, 0.41]	-0.19 [-0.64, 0.25]	-0.18 [-0.63, 0.29]	-0.22 [-0.69, 0.23]	49	34
trembling	-0.15 [-0.66, 0.33]	0.09 [-0.41, 0.55]	-0.14 [-0.63, 0.30]	-0.30 [-0.78, 0.17]	-0.24 [-0.75, 0.20]	46	31
pounding heart	0.18 [-0.30, 0.63]	0.14 [-0.33, 0.60]	-0.21 [-0.67, 0.22]	-0.37 [-0.86, 0.08]	-0.32 [-0.80, 0.13]	45	34
agitated	0.10 [-0.35, 0.55]	-0.19 [-0.62, 0.24]	-0.32 [-0.78, 0.10]	-0.12 [-0.58, 0.31]	-0.27 [-0.74, 0.17]	47	33
inner tension	0.12 [-0.34, 0.59]	0.24 [-0.21, 0.69]	-0.01 [-0.45, 0.44]	-0.27 [-0.73, 0.16]	-0.17 [-0.62, 0.26]	48	32

Supplementary Table 5. AUC values from item-level comparisons between peripheral and non-peripheral dizziness. Reported are ROC-derived AUC values with 95% confidence intervals for each item and measure of perceived intensity (see Figure 1). Bold item labels indicate that the AUC confidence interval lies entirely above or below 0.5 (including 0.5) for at least one measure. Shaded cells highlight such AUCs, with green indicating higher values in the peripheral group and blue in the non-peripheral group. Items are sorted according to Table 2.

Item	Self-Comparison	Dizziness – Calorics	Dizziness	Calorics	Dizziness + Calorics	n_1	n_2
**self spinning**	0.68 [0.56, 0.78]	0.78 [0.67, 0.87]	0.62 [0.50, 0.74]	0.27 [0.17, 0.40]	0.43 [0.30, 0.57]	48	33
**in head spinning**	0.72 [0.60, 0.82]	0.77 [0.65, 0.86]	0.65 [0.52, 0.76]	0.31 [0.20, 0.45]	0.46 [0.32, 0.59]	47	33
**world motion**	0.66 [0.55, 0.77]	0.66 [0.54, 0.78]	0.65 [0.53, 0.76]	0.40 [0.28, 0.53]	0.48 [0.35, 0.61]	48	32
**imbalance**	0.63 [0.52, 0.73]	0.74 [0.62, 0.84]	0.66 [0.55, 0.77]	0.31 [0.21, 0.44]	0.39 [0.27, 0.53]	48	32
**dizzy**	0.62 [0.51, 0.72]	0.72 [0.59, 0.82]	0.51 [0.40, 0.62]	0.29 [0.18, 0.42]	0.32 [0.20, 0.45]	49	33
**dazed**	0.54 [0.42, 0.65]	0.62 [0.49, 0.73]	0.46 [0.34, 0.58]	0.30 [0.20, 0.42]	0.34 [0.23, 0.47]	49	33
wrong in head	0.58 [0.47, 0.69]	0.56 [0.43, 0.68]	0.50 [0.37, 0.62]	0.43 [0.31, 0.56]	0.45 [0.32, 0.58]	48	33
**loss of surround**	0.61 [0.48, 0.72]	0.67 [0.54, 0.77]	0.53 [0.41, 0.66]	0.33 [0.22, 0.46]	0.41 [0.28, 0.55]	44	33
**loss of control**	0.54 [0.43, 0.66]	0.66 [0.53, 0.78]	0.56 [0.43, 0.68]	0.36 [0.25, 0.48]	0.43 [0.31, 0.57]	45	33
**confused**	0.56 [0.44, 0.67]	0.66 [0.54, 0.77]	0.52 [0.39, 0.64]	0.36 [0.26, 0.47]	0.44 [0.32, 0.58]	47	34
**empty brain**	0.47 [0.36, 0.60]	0.54 [0.42, 0.67]	0.44 [0.31, 0.57]	0.35 [0.25, 0.45]	0.40 [0.27, 0.53]	45	33
**near faint**	0.50 [0.38, 0.62]	0.50 [0.37, 0.62]	0.40 [0.28, 0.52]	0.38 [0.28, 0.48]	0.36 [0.25, 0.49]	47	34
not my body	0.46 [0.34, 0.57]	0.53 [0.40, 0.67]	0.42 [0.30, 0.55]	0.41 [0.29, 0.52]	0.38 [0.26, 0.53]	41	30
altered body	0.43 [0.31, 0.55]	0.49 [0.36, 0.62]	0.41 [0.28, 0.54]	0.44 [0.33, 0.54]	0.38 [0.26, 0.51]	43	31
slow motion	0.50 [0.39, 0.60]	0.42 [0.30, 0.53]	0.47 [0.34, 0.58]	0.54 [0.45, 0.62]	0.50 [0.37, 0.61]	47	31
sloshing in head	0.52 [0.40, 0.63]	0.49 [0.37, 0.61]	0.47 [0.35, 0.60]	0.49 [0.38, 0.61]	0.47 [0.34, 0.60]	43	32
wave motion	0.48 [0.36, 0.60]	0.53 [0.40, 0.65]	0.45 [0.32, 0.58]	0.44 [0.32, 0.55]	0.44 [0.31, 0.57]	47	31
sinking	0.54 [0.43, 0.65]	0.48 [0.35, 0.62]	0.43 [0.31, 0.56]	0.51 [0.40, 0.60]	0.44 [0.32, 0.58]	46	30
**neck dizziness**	0.39 [0.28, 0.52]	0.39 [0.27, 0.52]	0.38 [0.26, 0.50]	0.46 [0.34, 0.57]	0.37 [0.25, 0.50]	42	27
head pressure	0.50 [0.38, 0.62]	0.55 [0.42, 0.67]	0.51 [0.38, 0.64]	0.45 [0.33, 0.58]	0.48 [0.35, 0.62]	44	31
electric shock	0.46 [0.37, 0.54]	0.49 [0.39, 0.58]	0.44 [0.33, 0.53]	0.48 [0.41, 0.54]	0.44 [0.34, 0.53]	47	32
**heat rush**	0.66 [0.53, 0.76]	0.57 [0.44, 0.69]	0.47 [0.34, 0.59]	0.38 [0.27, 0.50]	0.42 [0.29, 0.56]	46	32
**sweating**	0.65 [0.54, 0.76]	0.62 [0.50, 0.74]	0.55 [0.43, 0.68]	0.39 [0.29, 0.50]	0.48 [0.35, 0.61]	47	32
**nauseous**	0.69 [0.58, 0.79]	0.75 [0.62, 0.84]	0.71 [0.59, 0.81]	0.41 [0.29, 0.54]	0.59 [0.46, 0.71]	49	34
anxious	0.42 [0.33, 0.53]	0.45 [0.33, 0.58]	0.48 [0.35, 0.60]	0.49 [0.37, 0.61]	0.47 [0.35, 0.60]	47	34
panic	0.50 [0.39, 0.62]	0.49 [0.36, 0.63]	0.44 [0.32, 0.57]	0.47 [0.36, 0.57]	0.43 [0.31, 0.56]	49	34
trembling	0.46 [0.34, 0.57]	0.51 [0.38, 0.63]	0.46 [0.33, 0.58]	0.43 [0.32, 0.55]	0.42 [0.29, 0.55]	46	31
pounding heart	0.53 [0.42, 0.65]	0.54 [0.41, 0.66]	0.44 [0.33, 0.58]	0.40 [0.28, 0.51]	0.40 [0.28, 0.53]	45	34
agitated	0.52 [0.40, 0.64]	0.45 [0.33, 0.58]	0.41 [0.29, 0.54]	0.45 [0.33, 0.58]	0.41 [0.29, 0.54]	47	33
inner tension	0.52 [0.41, 0.64]	0.56 [0.43, 0.69]	0.51 [0.38, 0.63]	0.42 [0.30, 0.55]	0.46 [0.33, 0.58]	48	32

2b) Comparison between peripheral and functional patient groups: group means and effect sizes (Cohen’s d and AUC values)

The following figure shows group mean comparisons analogous to Figure 1, but here peripheral patients are compared only with the functional subgroup rather than with all non-peripheral patients. The two subsequent tables, like the previous ones, present effect sizes (Cohen’s d and AUC values) for this comparison.

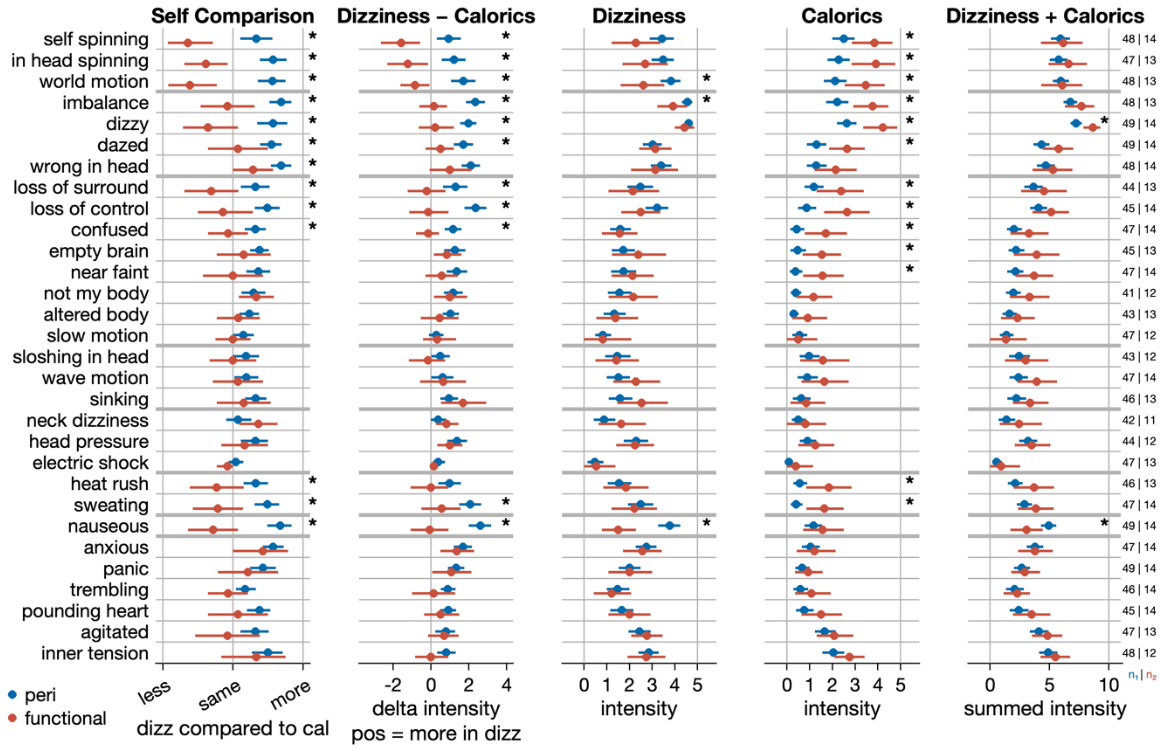

Supplementary Figure 1. Item-level comparison between peripheral and functional dizziness groups. Layout and interpretation follow that of Figure 1. The non-peripheral group was replaced by the subgroup of patients diagnosed with functional dizziness.

Supplementary Table 6. Effect sizes (Cohen's d) from item-level comparisons between peripheral and functional dizziness groups. Structure and interpretation follow that of Supplementary Table 4, except that the non-peripheral group was replaced by the functional group.

Item	Self-Comparison	Dizziness – Calorics	Dizziness	Calorics	Dizziness + Calorics	n_1	n_2
**self spinning**	1.26 [0.67, 2.01]	1.13 [0.60, 1.82]	0.59 [-0.03, 1.33]	-0.77 [-1.47, -0.14]	-0.07 [-0.71, 0.61]	48	14
**in head spinning**	1.42 [0.75, 2.37]	1.10 [0.50, 1.81]	0.45 [-0.18, 1.19]	-0.94 [-1.72, -0.27]	-0.31 [-1.01, 0.35]	47	13
**world motion**	1.68 [1.00, 2.78]	1.17 [0.68, 1.83]	0.74 [0.09, 1.58]	-0.76 [-1.42, -0.15]	-0.04 [-0.80, 0.69]	48	13
**imbalance**	1.22 [0.50, 2.27]	1.29 [0.81, 1.88]	0.70 [0.02, 1.61]	-0.91 [-1.59, -0.33]	-0.43 [-1.18, 0.25]	48	13
**dizzy**	1.19 [0.52, 2.16]	1.07 [0.38, 1.89]	0.29 [-0.45, 1.03]	-1.03 [-1.73, -0.40]	-0.84 [-1.45, -0.29]	49	14
**dazed**	0.75 [0.05, 1.57]	0.69 [0.20, 1.25]	-0.08 [-0.63, 0.50]	-0.86 [-1.57, -0.27]	-0.59 [-1.24, 0.01]	49	14
**wrong in head**	0.75 [0.11, 1.55]	0.60 [-0.06, 1.35]	0.14 [-0.51, 0.87]	-0.52 [-1.26, 0.11]	-0.21 [-0.90, 0.45]	48	14
**loss of surround**	0.84 [0.23, 1.62]	0.70 [0.15, 1.32]	0.16 [-0.48, 0.83]	-0.75 [-1.64, -0.05]	-0.31 [-1.08, 0.43]	44	13
**loss of control**	0.95 [0.30, 1.80]	1.25 [0.66, 2.00]	0.41 [-0.18, 1.07]	-1.14 [-2.16, -0.44]	-0.40 [-1.14, 0.26]	45	14
**confused**	0.69 [0.08, 1.35]	0.86 [0.40, 1.37]	0.02 [-0.58, 0.60]	-1.00 [-2.10, -0.28]	-0.51 [-1.35, 0.17]	47	14
**empty brain**	0.42 [-0.31, 1.16]	0.23 [-0.29, 0.74]	-0.34 [-1.12, 0.36]	-0.82 [-1.85, -0.11]	-0.64 [-1.53, 0.13]	45	13
**near faint**	0.56 [-0.10, 1.32]	0.42 [-0.12, 0.99]	-0.21 [-0.82, 0.35]	-0.98 [-2.04, -0.26]	-0.62 [-1.36, 0.04]	47	14
not my body	-0.07 [-0.70, 0.52]	0.10 [-0.56, 0.74]	-0.34 [-1.10, 0.32]	-0.79 [-1.81, 0.05]	-0.59 [-1.50, 0.18]	41	12
altered body	0.30 [-0.45, 1.02]	0.36 [-0.36, 1.02]	-0.03 [-0.71, 0.59]	-0.67 [-1.54, 0.20]	-0.30 [-1.05, 0.40]	43	13
slow motion	0.28 [-0.27, 0.83]	-0.04 [-0.74, 0.64]	-0.00 [-0.80, 0.80]	0.04 [-0.65, 0.60]	0.02 [-0.75, 0.76]	47	12
sloshing in head	0.30 [-0.32, 0.92]	0.39 [-0.28, 1.02]	0.03 [-0.62, 0.59]	-0.37 [-1.19, 0.34]	-0.18 [-0.89, 0.46]	43	12
wave motion	0.19 [-0.46, 0.88]	-0.01 [-0.65, 0.66]	-0.41 [-1.10, 0.20]	-0.44 [-1.21, 0.22]	-0.53 [-1.25, 0.10]	47	14
sinking	0.29 [-0.39, 1.01]	-0.42 [-1.23, 0.32]	-0.50 [-1.25, 0.16]	-0.16 [-0.89, 0.45]	-0.42 [-1.13, 0.19]	46	13
neck dizziness	-0.50 [-1.12, 0.07]	-0.34 [-1.10, 0.20]	-0.46 [-1.28, 0.22]	-0.28 [-1.16, 0.48]	-0.43 [-1.29, 0.31]	42	11
head pressure	0.23 [-0.33, 0.85]	0.23 [-0.30, 0.75]	0.03 [-0.56, 0.59]	-0.26 [-1.05, 0.41]	-0.11 [-0.80, 0.54]	44	12
electric shock	0.35 [-0.13, 0.84]	0.22 [-0.24, 0.48]	-0.05 [-0.77, 0.46]	-0.43 [-1.10, 0.40]	-0.20 [-0.93, 0.47]	47	13
**heat rush**	0.89 [0.21, 1.69]	0.47 [-0.09, 1.09]	-0.17 [-0.83, 0.44]	-0.98 [-2.00, -0.19]	-0.66 [-1.51, 0.11]	46	13
**sweating**	1.10 [0.47, 1.91]	0.74 [0.17, 1.39]	0.14 [-0.44, 0.75]	-1.12 [-2.11, -0.38]	-0.40 [-1.11, 0.28]	47	14
**nauseous**	1.48 [0.78, 2.61]	1.26 [0.66, 2.13]	1.33 [0.71, 2.26]	-0.27 [-0.97, 0.37]	0.78 [0.15, 1.56]	49	14
anxious	0.25 [-0.46, 0.99]	0.20 [-0.39, 0.84]	0.11 [-0.51, 0.76]	-0.12 [-0.78, 0.53]	0.00 [-0.65, 0.66]	47	14
panic	0.31 [-0.32, 1.03]	0.15 [-0.54, 0.87]	0.00 [-0.64, 0.63]	-0.22 [-0.88, 0.35]	-0.10 [-0.71, 0.46]	49	14
trembling	0.47 [-0.20, 1.17]	0.46 [-0.31, 1.22]	0.16 [-0.44, 0.72]	-0.39 [-1.15, 0.25]	-0.09 [-0.70, 0.43]	46	14
pounding heart	0.48 [-0.22, 1.26]	0.27 [-0.41, 0.99]	-0.19 [-0.85, 0.41]	-0.51 [-1.29, 0.13]	-0.38 [-1.09, 0.23]	45	14
agitated	0.52 [-0.12, 1.31]	0.05 [-0.49, 0.66]	-0.19 [-0.75, 0.34]	-0.26 [-0.90, 0.32]	-0.27 [-0.87, 0.28]	47	13
inner tension	0.22 [-0.44, 0.98]	0.47 [-0.13, 1.11]	0.07 [-0.54, 0.71]	-0.43 [-0.99, 0.05]	-0.22 [-0.80, 0.32]	48	12

Supplementary Table 7. AUC values from item-level comparisons between peripheral and functional dizziness. Structure and interpretation follow that of Supplementary Table 5, except that the non-peripheral group was replaced by the functional group.

Item	Self-Comparison	Dizziness – Calorics	Dizziness	Calorics	Dizziness + Calorics	n_1	n_2
**self spinning**	0.83 [0.68, 0.91]	0.79 [0.62, 0.90]	0.63 [0.44, 0.79]	0.24 [0.11, 0.49]	0.40 [0.22, 0.61]	47	13
**in head spinning**	0.86 [0.71, 0.94]	0.83 [0.69, 0.91]	0.70 [0.52, 0.84]	0.30 [0.16, 0.49]	0.46 [0.28, 0.68]	48	13
**world motion**	0.80 [0.65, 0.90]	0.81 [0.66, 0.90]	0.66 [0.48, 0.80]	0.27 [0.13, 0.47]	0.47 [0.27, 0.67]	48	14
**imbalance**	0.76 [0.59, 0.87]	0.69 [0.52, 0.82]	0.54 [0.36, 0.69]	0.26 [0.13, 0.43]	0.39 [0.21, 0.58]	47	14
**dizzy**	0.71 [0.52, 0.84]	0.59 [0.43, 0.75]	0.44 [0.27, 0.61]	0.32 [0.16, 0.50]	0.35 [0.18, 0.58]	46	13
**dazed**	0.63 [0.45, 0.78]	0.59 [0.42, 0.75]	0.42 [0.27, 0.58]	0.29 [0.15, 0.45]	0.34 [0.19, 0.52]	47	14
**wrong in head**	0.68 [0.52, 0.81]	0.65 [0.44, 0.80]	0.51 [0.33, 0.70]	0.37 [0.21, 0.55]	0.44 [0.26, 0.63]	48	14
**loss of surround**	0.72 [0.53, 0.84]	0.69 [0.51, 0.82]	0.55 [0.34, 0.72]	0.33 [0.16, 0.53]	0.44 [0.24, 0.65]	44	13
loss of control	0.63 [0.44, 0.80]	0.52 [0.34, 0.69]	0.45 [0.29, 0.61]	0.41 [0.26, 0.59]	0.41 [0.25, 0.59]	47	13
**confused**	0.66 [0.48, 0.81]	0.68 [0.53, 0.81]	0.49 [0.32, 0.66]	0.27 [0.15, 0.42]	0.33 [0.18, 0.52]	49	14
empty brain	0.57 [0.41, 0.71]	0.55 [0.38, 0.71]	0.50 [0.33, 0.67]	0.44 [0.25, 0.62]	0.47 [0.27, 0.65]	44	12
near faint	0.56 [0.38, 0.73]	0.44 [0.24, 0.65]	0.36 [0.20, 0.56]	0.46 [0.29, 0.59]	0.36 [0.21, 0.55]	46	13
not my body	0.60 [0.44, 0.74]	0.61 [0.41, 0.75]	0.55 [0.37, 0.69]	0.41 [0.25, 0.56]	0.46 [0.30, 0.64]	46	14
altered body	0.38 [0.23, 0.52]	0.36 [0.21, 0.51]	0.37 [0.20, 0.54]	0.47 [0.27, 0.61]	0.39 [0.21, 0.57]	42	11
slow motion	0.54 [0.36, 0.70]	0.50 [0.33, 0.68]	0.38 [0.22, 0.55]	0.41 [0.24, 0.56]	0.36 [0.20, 0.53]	47	14
sloshing in head	0.54 [0.38, 0.70]	0.57 [0.39, 0.73]	0.54 [0.35, 0.71]	0.47 [0.30, 0.64]	0.51 [0.33, 0.69]	47	14
wave motion	0.57 [0.43, 0.68]	0.51 [0.35, 0.65]	0.58 [0.34, 0.70]	0.52 [0.35, 0.62]	0.57 [0.35, 0.70]	47	12
**sinking**	0.56 [0.50, 0.68]	0.50 [0.35, 0.58]	0.46 [0.30, 0.57]	0.49 [0.34, 0.55]	0.47 [0.31, 0.58]	47	13
**neck dizziness**	0.72 [0.54, 0.85]	0.81 [0.65, 0.91]	0.63 [0.45, 0.77]	0.24 [0.11, 0.42]	0.38 [0.20, 0.59]	45	14
head pressure	0.49 [0.32, 0.63]	0.53 [0.34, 0.71]	0.41 [0.23, 0.60]	0.38 [0.19, 0.54]	0.37 [0.18, 0.58]	41	12
electric shock	0.60 [0.41, 0.77]	0.59 [0.39, 0.75]	0.45 [0.28, 0.61]	0.35 [0.20, 0.51]	0.38 [0.22, 0.56]	45	14
**heat rush**	0.77 [0.60, 0.89]	0.82 [0.68, 0.91]	0.67 [0.50, 0.82]	0.25 [0.13, 0.43]	0.34 [0.19, 0.53]	48	13
**sweating**	0.77 [0.62, 0.88]	0.78 [0.58, 0.90]	0.53 [0.39, 0.71]	0.21 [0.10, 0.41]	0.26 [0.15, 0.44]	49	14
**nauseous**	0.82 [0.67, 0.92]	0.83 [0.70, 0.92]	0.82 [0.66, 0.91]	0.44 [0.26, 0.62]	0.71 [0.51, 0.85]	49	14
**anxious**	0.66 [0.50, 0.78]	0.70 [0.54, 0.83]	0.50 [0.32, 0.67]	0.29 [0.15, 0.46]	0.40 [0.21, 0.60]	47	14
panic	0.55 [0.39, 0.73]	0.65 [0.44, 0.81]	0.53 [0.33, 0.71]	0.37 [0.23, 0.52]	0.44 [0.28, 0.63]	48	12
trembling	0.57 [0.40, 0.73]	0.56 [0.35, 0.75]	0.50 [0.31, 0.67]	0.42 [0.27, 0.57]	0.46 [0.30, 0.63]	49	14
**pounding heart**	0.58 [0.40, 0.75]	0.56 [0.39, 0.72]	0.42 [0.23, 0.62]	0.32 [0.17, 0.48]	0.38 [0.19, 0.59]	45	13
agitated	0.58 [0.41, 0.72]	0.55 [0.37, 0.71]	0.50 [0.32, 0.67]	0.42 [0.24, 0.61]	0.45 [0.27, 0.65]	43	12
inner tension	0.56 [0.38, 0.71]	0.59 [0.39, 0.74]	0.50 [0.32, 0.66]	0.42 [0.24, 0.56]	0.44 [0.26, 0.62]	43	13

2c) Explaining the advantage of symptom comparison: discrimination strength based on AUC values

Visual inspection of group mean differences (Figure 1 and Supplementary Figure 1) suggests that comparing symptoms between self-experienced dizziness and caloric stimulation may offer a diagnostic advantage over assessing self-experienced dizziness symptoms alone. To explore this further and better understand the source of this advantage, the following two figures present results based on AUC values as a measure of discrimination strength. For each symptom, two symptom measures were compared per column to assess whether one showed superior discrimination performance. The first figure displays the comparison between peripheral and non-peripheral patients; the second shows the comparison with the functional subgroup.

In the first column of both figures, the advantage of the symptom comparison over self-experienced dizziness symptoms is directly visible as the difference in AUC values (highlighted in green). In line with the pattern observed in the group mean differences, and particularly in the comparison with the functional subgroup, the symptom comparison appears to offer a diagnostic advantage over assessing self-experienced dizziness symptoms alone for spinning vertigo, imbalance, general dizziness symptoms, certain depersonalisation/derealisation symptoms ("loss of surround", "loss of control", and "confused"), sweating, and, to some extent, nausea.

Given the reverse pattern observed in the group mean differences for spinning vertigo, imbalance, and nausea, one would expect the symptom comparison to outperform not only the self-experienced dizziness ratings (column 1) but also the caloric ratings (column 2) for these symptoms. The results appear to support this expectation, although the confidence intervals include zero. As additionally shown in column 3, caloric stimulation appears to contribute more strongly than self-experienced dizziness, except for the symptom "world motion" and, most notably, for "nausea," where the group discrimination achieved through the symptom comparison is primarily attributable to differences in self-experienced dizziness.

In contrast, based on the pattern of group mean differences for general dizziness symptoms, certain depersonalisation/derealisation symptoms ("loss of surround", "loss of control", and "confused"), and sweating, the advantage of the symptom comparison was expected to be driven primarily by the caloric condition, with no additional benefit over caloric ratings alone.

This expectation is supported by the fact that the AUC differences (column 2) are close to zero on average, and that caloric stimulation discriminates clearly better than self-experienced dizziness alone (column 3).

The notion that the summed intensity across both conditions could provide an advantage over caloric symptoms alone (column 4) finds little support. At best, this might apply to symptoms such as “neck dizziness” or “sinking.”

Regarding the question of whether self-comparison ratings offer added value over calculated difference scores, the results are inconclusive (column 5). In the comparison between peripheral and non-peripheral patients, self-comparison appears to be less informative (highlighted in blue) for nearly all of the previously identified relevant symptoms. However, when comparing peripheral patients with the functional subgroup, no meaningful difference is apparent. Notably, when examining mean self-comparison ratings within the functional subgroup, several symptoms were perceived as more intense during caloric stimulation than suggested by the direct difference scores (see Supplementary Figure 1). Thus, it remains unclear which of the two approaches more accurately reflects perceived symptom differences.

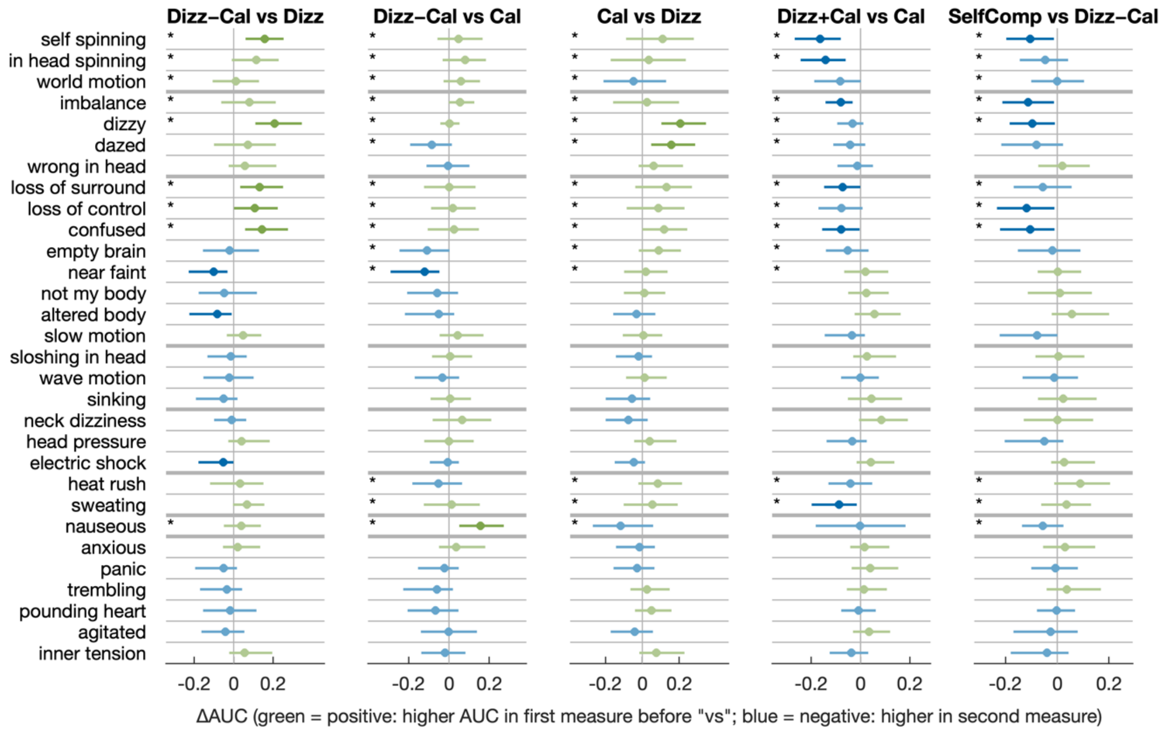

Supplementary Figure 2. Discriminative value of symptom measures: pairwise comparisons of AUC values for distinguishing peripheral vs. non-peripheral dizziness. For each item, ΔAUC values and their 95% confidence intervals are shown for five different pairwise comparisons of symptom measures. In each comparison, the AUC of the second-listed measure in the subplot title was subtracted from that of the first-listed measure. Color shading indicates whether the resulting ΔAUC value is positive or negative. Bold lines indicate that the 95% CI does not include zero. Asterisks denote cases in which the 95% CI of at least one of the two individual AUCs lies entirely above or below 0.5 (including 0.5). Abbreviations: Cal = calorics, Dizz = dizziness, SelfComp = self-comparison.

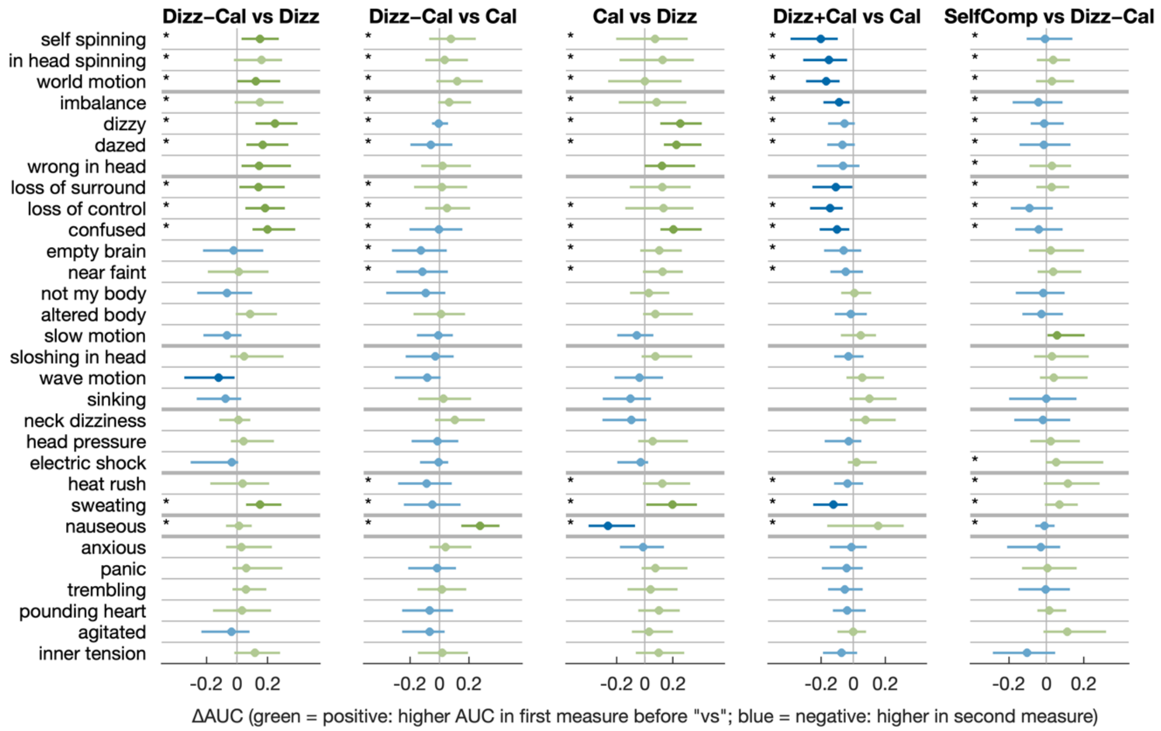

Supplementary Figure 3. Discriminative value of symptom measures: pairwise comparisons of AUC values for distinguishing peripheral vs. functional dizziness. Layout and interpretation follow that of Supplementary Figure 2. The non-peripheral group was replaced by the subgroup of patients diagnosed with functional dizziness.

3) Caloric symptoms are not explained by differences in slow-phase velocity

A potential explanation for group differences in caloric symptoms is variation in caloric excitability, as measured by the slow-phase velocity (SPV) of caloric nystagmus. However, maximum SPV did not differ between groups. Across all four stimulations (cold/warm water, left/right ear), it was 21.9 °/s (SD = 11.3) in the peripheral group and 22.5 °/s (SD = 8.3) in the non-peripheral group (Cohen's d = –0.06, 95% CI [–0.54, 0.33]).

Despite being instructed to report their subjective experience during the strongest caloric stimulation, average excitability (as reflected by mean SPV) may be more closely related to the reported symptom intensity than the maximum SPV. Mean SPV differed between groups: the peripheral group showed lower values (M = 13.0 °/s, SD = 6.6) than the non-peripheral group (M = 16.1 °/s, SD = 6.1; Cohen's d = –0.48, 95% CI [–0.98, –0.06]). To account for this, we formed two subgroups by including only patients whose mean SPV exceeded a predefined threshold, set at the median of the original non-peripheral group (14.25 °/s). In these matched subgroups (n_1 = 17, n_2 = 18), mean SPV was nearly identical: 20.7 °/s (SD = 4.8) in the peripheral group and 20.9 °/s (SD = 4.2) in the non-peripheral group (Cohen's d = –0.05, 95% CI [–0.75, 0.63]). Supplementary Figure 4 shows the group mean differences in caloric symptoms for the SPV-matched subgroups compared to the full sample. No meaningful differences were observed.

Given the difference observed in the intensity of caloric symptoms between patients with peripheral dizziness and those without dizziness, we also examined potential group differences in caloric excitability. No such differences were found: Mean SPV was 15.6 °/s (SD = 7.7) in the non-dizzy group and 13.0 °/s (SD = 6.6) in the peripheral group (Cohen's d = 0.37, 95% CI [–0.16, 0.93]). Maximum SPV was 23.6 °/s versus 21.9 °/s, with a Cohen's d of 0.15 (95% CI [–0.34, 0.71]).

Thus, there is a dissociation between caloric excitability, as reflected by SPV, and the symptoms elicited by caloric stimulation.

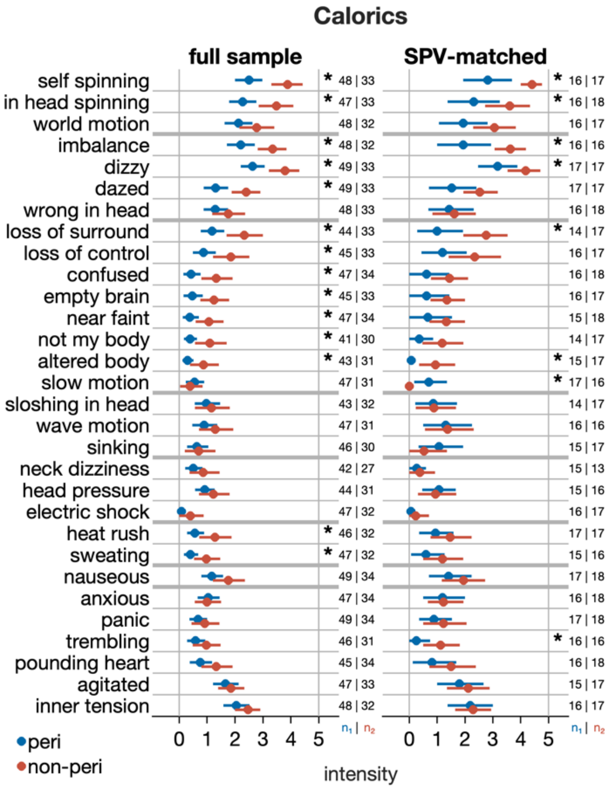

Supplementary Figure 4. Group differences in symptom ratings during caloric stimulation: full sample vs. SPV-matched subsample. For each item, perceived intensities are shown as group means (peripheral vs. non-peripheral dizziness) with 95% confidence intervals, as in Figure 1. The left column reproduces the full sample results (identical to column 4 in Figure 1); the right column shows the SPV-matched subsample.

4) Questionnaires

4a) Dizziness Questionnaire

	Wie war Ihr Gefühl **während Ihres Schwindels**? Bitte kreuzen Sie an:						1
**Ich habe…**	0 gar nicht	1 sehr leicht	2 leicht	3 mittel	4 stark	5 sehr stark	weiß nicht
… Übelkeit verspürt.	○	○	○	○	○	○	○
… mich schwindelig gefühlt.	○	○	○	○	○	○	○
… mich aufgeregt oder nervös gefühlt.	○	○	○	○	○	○	○
… das Gefühl gehabt, mich nach rechts oder links zu drehen (wie im Karussell).	○	○	○	○	○	○	○
… das Gefühl gehabt, dass es mir plötzlich wärmer wird oder dass mich ein Hitzegefühl durchläuft.	○	○	○	○	○	○	○
… das Gefühl gehabt, dass mit meinem Gleichgewicht irgendwas nicht stimmt.	○	○	○	○	○	○	○
… ein Gefühl gehabt, als ob ein elektrischer Schlag durch meinen Kopf fährt.	○	○	○	○	○	○	○
… den Eindruck gehabt, den Bezug zur Umgebung zu verlieren (z.B. nicht mehr richtig zu wissen, wo oben und unten ist).	○	○	○	○	○	○	○
… bemerkt, dass bestimmte Körperregionen (z.B. Hände oder Füße) gezittert haben.	○	○	○	○	○	○	○
… das Gefühl gehabt, als würde ich gleich in Ohnmacht fallen.	○	○	○	○	○	○	○

	Wie war Ihr Gefühl **während Ihres Schwindels**? Bitte kreuzen Sie an:						2
**Ich habe…**	0 gar nicht	1 sehr leicht	2 leicht	3 mittel	4 stark	5 sehr stark	weiß nicht
… mich verwirrt gefühlt.	○	○	○	○	○	○	○
… Angst verspürt.	○	○	○	○	○	○	○
… ein Druckgefühl im Kopf verspürt.	○	○	○	○	○	○	○
… den Eindruck gehabt, dass sich die Umgebung um mich herum bewegt.	○	○	○	○	○	○	○
… geschwitzt.	○	○	○	○	○	○	○
… mich innerlich angespannt gefühlt.	○	○	○	○	○	○	○
… das Gefühl gehabt, dass Teile meines Körpers oder mein ganzer Körper nicht so richtig zu mir gehören.	○	○	○	○	○	○	○
… den Eindruck gehabt, dass sich mein Körper auf und ab bewegt (wie von einer Welle auf dem Meer getragen oder wie auf einem Boot bei starkem Wellengang).	○	○	○	○	○	○	○
… den Eindruck gehabt, dass alles wie in Zeitlupe abläuft.	○	○	○	○	○	○	○
… das Gefühl gehabt abzusacken (der Situation vergleichbar, bei der man im Flugzeug sitzt und Turbulenzen auftreten).	○	○	○	○	○	○	○

	Wie war Ihr Gefühl **während Ihres Schwindels**? Bitte kreuzen Sie an:						3
**Ich habe…**	0 gar nicht	1 sehr leicht	2 leicht	3 mittel	4 stark	5 sehr stark	weiß nicht
… Herzklopfen oder Herzrasen gehabt.	○	○	○	○	○	○	○
… mich wie benommen gefühlt.	○	○	○	○	○	○	○
… regelrecht Panik gehabt.	○	○	○	○	○	○	○
… das Gefühl gehabt, dass sich etwas in meinem Kopf dreht.	○	○	○	○	○	○	○
… irgendwie keine richtige Kontrolle mehr über meinen Körper gehabt.	○	○	○	○	○	○	○
… die Empfindung gehabt, dass ein Schwindel, vom Nacken hochkommt.	○	○	○	○	○	○	○
… das Gefühl gehabt, dass in meinem Kopf irgendwas nicht stimmt.	○	○	○	○	○	○	○
… das Gefühl gehabt, keinen klaren Gedanken fassen zu können, fast als ob mein Gehirn wie leergefegt wäre.	○	○	○	○	○	○	○
… den Eindruck gehabt, dass in meinem Kopf etwas hin und her schwappt.	○	○	○	○	○	○	○
… das Gefühl gehabt, dass sich Teile meines Körpers oder sogar mein ganzer Körper anders anfühlen (z.B. wie taub, pelzig oder kribbelig).	○	○	○	○	○	○	○

4b) Caloric-and-Self-Comparison Questionnaire

Fragebogen Vergleich

1) Mir war bei der Untersuchung gerade übel.						
0<br>nein, gar nicht<br>○	1<br>sehr leicht<br>○	2<br>leicht<br>○	3<br>mittel<br>○	4<br>stark<br>○	5<br>sehr stark<br>○	weiß nicht<br>○

2) Bei meinem Schwindel(anfall) war mir ...				
nicht übel<br>○	weniger übel<br>als bei der Untersuchung<br>○	stärker übel<br>als bei der Untersuchung<br>○	genauso übel<br>wie bei der Untersuchung<br>○	weiß nicht<br>○

3) Mir war bei der Untersuchung gerade schwindelig.						
0<br>nein, gar nicht<br>○	1<br>sehr leicht<br>○	2<br>leicht<br>○	3<br>mittel<br>○	4<br>stark<br>○	5<br>sehr stark<br>○	weiß nicht<br>○

4) Bei meinem Schwindel(anfall) war mir ...				
nicht schwindelig<br>○	weniger schwindelig<br>als bei der Untersuchung<br>○	stärker schwindelig<br>als bei der Untersuchung<br>○	genauso schwindelig<br>wie bei der Untersuchung<br>○	weiß nicht<br>○

5) Ich war bei der Untersuchung gerade aufgeregt oder nervös.						
0<br>nein, gar nicht<br>○	1<br>sehr leicht<br>○	2<br>leicht<br>○	3<br>mittel<br>○	4<br>stark<br>○	5<br>sehr stark<br>○	weiß nicht<br>○

6) Bei meinem Schwindel(anfall) war ich ...				
nicht aufgeregt<br>oder nervös<br>○	weniger aufgeregt oder<br>nervös<br>als bei der Untersuchung<br>○	stärker aufgeregt oder<br>nervöser<br>als bei der Untersuchung<br>○	genauso aufgeregt oder<br>nervös<br>wie bei der Untersuchung<br>○	weiß nicht<br>○

7) Ich habe bei der Untersuchung gerade das Gefühl gehabt, mich nach rechts oder links zu drehen (wie im Karussell).						
0<br>nein, gar nicht<br>○	1<br>sehr leicht<br>○	2<br>leicht<br>○	3<br>mittel<br>○	4<br>stark<br>○	5<br>sehr stark<br>○	weiß nicht<br>○

8) Bei meinem Schwindel(anfall) habe ich mich ...				
nicht gedreht wie<br>im Karussell<br>○	weniger gedreht als bei<br>der Untersuchung<br>○	stärker gedreht<br>als bei der Untersuchung<br>○	Genauso gedreht<br>wie bei der Untersuchung<br>○	weiß nicht<br>○

9) Ich habe bei der Untersuchung gerade das Gefühl gehabt, dass mir plötzlich wärmer wird oder mich ein Hitzegefühl durchläuft.						
0<br>nein, gar nicht<br>○	1<br>sehr leicht<br>○	2<br>leicht<br>○	3<br>mittel<br>○	4<br>stark<br>○	5<br>sehr stark<br>○	weiß nicht<br>○

10) Bei meinem Schwindel(anfall) hatte ich ...				
so ein Hitzegefühl<br>nicht<br>○	so ein Hitzegefühl weniger<br>als bei der Untersuchung<br>○	so ein Hitzegefühl stärker<br>als bei der Untersuchung<br>○	so ein Hitzegefühl genauso<br>stark<br>wie bei der Untersuchung<br>○	weiß nicht<br>○

11) Ich habe bei der Untersuchung gerade das Gefühl gehabt, dass mit meinem Gleichgewicht irgendwas nicht stimmt.						
0<br>nein, gar nicht<br>◯	1<br>sehr leicht<br>◯	2<br>leicht<br>◯	3<br>mittel<br>◯	4<br>stark<br>◯	5<br>sehr stark<br>◯	weiß nicht<br>◯

12) Bei meinem Schwindelanfall war mein…				
Gleichgewicht nicht gestört<br>◯	Gleichgewicht weniger gestört als bei der Untersuchung<br>◯	Gleichgewicht stärker gestört als bei der Untersuchung<br>◯	Gleichgewicht genauso stark gestört wie bei der Untersuchung<br>◯	weiß nicht<br>◯

13) Ich habe bei der Untersuchung gerade das Gefühl gehabt, als ob ein elektrischer Schlag durch meinen Kopf fährt.						
0<br>nein, gar nicht<br>◯	1<br>sehr leicht<br>◯	2<br>leicht<br>◯	3<br>mittel<br>◯	4<br>stark<br>◯	5<br>sehr stark<br>◯	weiß nicht<br>◯

14) Bei meinem Schwindel(anfall) hatte ich …				
so einen elektrischen Schlag nicht<br>◯	so einen elektrischen Schlag weniger als bei der Untersuchung<br>◯	So einen elektrischen Schlag stärker als bei der Untersuchung<br>◯	So einen elektrischen Schlag genauso stark wie bei der Untersuchung<br>◯	weiß nicht<br>◯

15) Ich habe bei der Untersuchung gerade den Eindruck gehabt, den Bezug zur Umgebung zu verlieren (z.B. nicht mehr richtig zu wissen, wo oben und unten ist).						
0<br>nein, gar nicht<br>◯	1<br>sehr leicht<br>◯	2<br>leicht<br>◯	3<br>mittel<br>◯	4<br>stark<br>◯	5<br>sehr stark<br>◯	weiß nicht<br>◯

16) Bei meinem Schwindel(anfall) habe ich…				
nicht den Bezug zur Umgebung verloren<br>◯	habe ich weniger den Bezug zur Umgebung verloren als bei der Untersuchung<br>◯	habe ich stärker den Bezug zur Umgebung verloren als bei der Untersuchung<br>◯	genauso stark den Bezug zur Umgebung verloren wie bei der Untersuchung<br>◯	weiß nicht<br>◯

17) Ich habe bei der Untersuchung gerade bemerkt, dass bestimmte Körperregionen (z.B. Hände oder Füße) gezittert haben.						
0<br>nein, gar nicht<br>◯	1<br>sehr leicht<br>◯	2<br>leicht<br>◯	3<br>mittel<br>◯	4<br>stark<br>◯	5<br>sehr stark<br>◯	weiß nicht<br>◯

18) Bei meinem Schwindel(anfall) hatte ich …				
so ein Zittern nicht<br>◯	so ein Zittern weniger als bei der Untersuchung<br>◯	so ein Zittern stärker als bei der Untersuchung<br>◯	so ein Zittern genauso stark wie bei der Untersuchung<br>◯	weiß nicht<br>◯

19) Ich habe bei der Untersuchung gerade das Gefühl gehabt, als würde ich gleich in Ohnmacht fallen.						
0<br>nein, gar nicht<br>◯	1<br>sehr leicht<br>◯	2<br>leicht<br>◯	3<br>mittel<br>◯	4<br>stark<br>◯	5<br>sehr stark<br>◯	weiß nicht<br>◯

20) Bei meinem Schwindel(anfall) hatte ich …				
so ein Ohnmachtsgefühl nicht<br>◯	So ein Ohnmachtsgefühl weniger als bei der Untersuchung<br>◯	So ein Ohnmachtsgefühl stärker als bei der Untersuchung<br>◯	So ein Ohnmachtsgefühl genauso stark wie bei der Untersuchung<br>◯	weiß nicht<br>◯

21) Ich habe mich bei der Untersuchung gerade verwirrt gefühlt.						
0<br>nein, gar nicht<br>○	1<br>sehr leicht<br>○	2<br>leicht<br>○	3<br>mittel<br>○	4<br>stark<br>○	5<br>sehr stark<br>○	weiß nicht<br>○

22) Bei meinem Schwindel(anfall) war ich …				
nicht verwirrt<br>○	weniger verwirrt als bei der Untersuchung<br>○	stärker verwirrt als bei der Untersuchung<br>○	genauso stark verwirrt wie bei der Untersuchung<br>○	weiß nicht<br>○

23) Ich habe bei der Untersuchung gerade Angst verspürt.						
0<br>nein, gar nicht<br>○	1<br>sehr leicht<br>○	2<br>leicht<br>○	3<br>mittel<br>○	4<br>stark<br>○	5<br>sehr stark<br>○	weiß nicht<br>○

24) Bei meinem Schwindel(anfall) hatte ich…				
keine Angst<br>○	weniger Angst als bei der Untersuchung<br>○	stärkere Angst als bei der Untersuchung<br>○	genauso starke Angst wie bei der Untersuchung<br>○	weiß nicht<br>○

25) Ich habe bei der Untersuchung gerade ein Druckgefühl im Kopf verspürt.						
0<br>nein, gar nicht<br>○	1<br>sehr leicht<br>○	2<br>leicht<br>○	3<br>mittel<br>○	4<br>stark<br>○	5<br>sehr stark<br>○	weiß nicht<br>○

26) Bei meinem Schwindel(anfall) hatte ich …				
so ein Druckgefühl nicht<br>○	so ein Druckgefühl weniger als bei der Untersuchung<br>○	so ein Druckgefühl stärker als bei der Untersuchung<br>○	so ein Druckgefühl genauso stark wie bei der Untersuchung<br>○	weiß nicht<br>○

27) Ich habe bei der Untersuchung gerade den Eindruck gehabt, dass sich die Umgebung um mich herum bewegt.						
0<br>nein, gar nicht<br>○	1<br>sehr leicht<br>○	2<br>leicht<br>○	3<br>mittel<br>○	4<br>stark<br>○	5<br>sehr stark<br>○	weiß nicht<br>○

28) Bei meinem Schwindel(anfall) hat sich…				
die Umgebung nicht bewegt<br>○	die Umgebung weniger bewegt als bei der Untersuchung<br>○	die Umgebung stärker bewegt als bei der Untersuchung<br>○	die Umgebung genauso stark bewegt wie bei der Untersuchung<br>○	weiß nicht<br>○

29) Ich habe bei der Untersuchung gerade geschwitzt.						
0<br>nein, gar nicht<br>○	1<br>sehr leicht<br>○	2<br>leicht<br>○	3<br>mittel<br>○	4<br>stark<br>○	5<br>sehr stark<br>○	weiß nicht<br>○

30) Bei meinem Schwindel(anfall) habe ich …				
nicht geschwitzt<br>○	weniger geschwitzt als bei der Untersuchung<br>○	stärker geschwitzt als bei der Untersuchung<br>○	genauso stark geschwitzt wie bei der Untersuchung<br>○	weiß nicht<br>○

31) Ich habe mich bei der Untersuchung gerade innerlich angespannt gefühlt.						
0<br>nein, gar nicht<br>◯	1<br>sehr leicht<br>◯	2<br>leicht<br>◯	3<br>mittel<br>◯	4<br>stark<br>◯	5<br>sehr stark<br>◯	weiß nicht<br>◯

32) Bei meinem Schwindel(anfall) war ich...				
nicht innerlich angespannt<br>◯	weniger angespannt als bei der Untersuchung<br>◯	stärker angespannt als bei der Untersuchung<br>◯	genauso stark angespannt wie bei der Untersuchung<br>◯	weiß nicht<br>◯

33) Ich habe bei der Untersuchung gerade das Gefühl gehabt, dass Teile meines Körpers oder mein ganzer Körper nicht so richtig zu mir gehören.						
0<br>nein, gar nicht<br>◯	1<br>sehr leicht<br>◯	2<br>leicht<br>◯	3<br>mittel<br>◯	4<br>stark<br>◯	5<br>sehr stark<br>◯	weiß nicht<br>◯

34) Bei meinem Schwindel(anfall) hatte ich ...				
dieses Gefühl nicht<br>◯	dieses Gefühl weniger als bei der Untersuchung<br>◯	dieses Gefühl stärker als bei der Untersuchung<br>◯	dieses Gefühl genauso stark wie bei der Untersuchung<br>◯	weiß nicht<br>◯

35) Ich habe bei der Untersuchung gerade den Eindruck gehabt, dass sich mein Körper auf und ab bewegt (wie von einer Welle auf dem Meer getragen oder wie auf einem Boot bei starkem Wellengang).						
0<br>nein gar nicht<br>◯	1<br>sehr leicht<br>◯	2<br>leicht<br>◯	3<br>mittel<br>◯	4<br>stark<br>◯	5<br>sehr stark<br>◯	weiß nicht<br>◯

36) Bei meinem Schwindel(anfall) hatte ich...				
nicht das Gefühl wie von einer Welle getragen zu werden<br>◯	das Gefühl wie von einer Welle getragen zu werden weniger als bei der Untersuchung<br>◯	das Gefühl wie von einer Welle getragen zu werden stärker als bei der Untersuchung<br>◯	das Gefühl wie von einer Welle getragen zu werden genauso stark wie bei der Untersuchung<br>◯	weiß nicht<br>◯

37) Ich habe bei der Untersuchung gerade den Eindruck gehabt, dass alles wie in Zeitlupe abläuft.						
0<br>nein, gar nicht<br>◯	1<br>sehr leicht<br>◯	2<br>leicht<br>◯	3<br>mittel<br>◯	4<br>stark<br>◯	5<br>sehr stark<br>◯	weiß nicht<br>◯

38) Bei meinem Schwindel(anfall) hatte ich ...				
nicht das Gefühl, dass alles wie in Zeitlupe abläuft<br>◯	weniger als bei der Untersuchung das Gefühl, dass alles wie in Zeitlupe abläuft<br>◯	stärker als bei der Untersuchung das Gefühl, dass alles wie in Zeitlupe abläuft<br>◯	genauso stark wie bei der Untersuchung das Gefühl, dass alles wie in Zeitlupe abläuft<br>◯	weiß nicht<br>◯

39) Ich habe bei der Untersuchung gerade das Gefühl gehabt abzusacken (der Situation vergleichbar, bei der man im Flugzeug sitzt und Turbulenzen auftreten).						
0<br>gar nicht<br>◯	1<br>sehr leicht<br>◯	2<br>leicht<br>◯	3<br>mittel<br>◯	4<br>stark<br>◯	5<br>sehr stark<br>◯	weiß nicht<br>◯

40) Bei meinem Schwindel(anfall) hatte ich...				
nicht das Gefühl abzusacken<br>◯	das Gefühl abzusacken weniger als bei der Untersuchung<br>◯	das Gefühl abzusacken stärker als bei der Untersuchung<br>◯	das Gefühl abzusacken genauso stark wie bei der Untersuchung<br>◯	weiß nicht<br>◯

41) Ich habe bei der Untersuchung gerade Herzklopfen oder Herzrasen gehabt.						
0<br>nein, gar nicht<br>○	1<br>sehr leicht<br>○	2<br>leicht<br>○	3<br>mittel<br>○	4<br>stark<br>○	5<br>sehr stark<br>○	weiß nicht<br>○

42) Bei meinem Schwindel(anfall) hatte ich …				
kein Herzklopfen oder Herzrasen<br>○	weniger Herzklopfen oder Herzrasen als bei der Untersuchung<br>○	stärkeres Herzklopfen oder Herzrasen als bei der Untersuchung<br>○	genauso starkes Herzklopfen oder -rasen wie bei der Untersuchung<br>○	weiß nicht<br>○

43) Ich habe mich bei der Untersuchung gerade wie benommen gefühlt.						
0<br>nein, gar nicht<br>○	1<br>sehr leicht<br>○	2<br>leicht<br>○	3<br>mittel<br>○	4<br>stark<br>○	5<br>sehr stark<br>○	weiß nicht<br>○

44) Bei meinem Schwindel(anfall) war ich …				
nicht wie benommen<br>○	weniger benommen als bei der Untersuchung<br>○	stärker benommen als bei der Untersuchung<br>○	genauso stark benommen wie bei der Untersuchung<br>○	weiß nicht<br>○

45) Ich habe bei der Untersuchung gerade regelrecht Panik gehabt.						
0<br>nein, gar nicht<br>○	1<br>sehr leicht<br>○	2<br>leicht<br>○	3<br>mittel<br>○	4<br>stark<br>○	5<br>sehr stark<br>○	weiß nicht<br>○

46) Bei meinem Schwindel(anfall) hatte …				
ich keine Panik<br>○	weniger Panik als bei der Untersuchung<br>○	stärker Panik als bei der Untersuchung<br>○	genauso stark Panik wie bei der Untersuchung<br>○	weiß nicht<br>○

47) Ich habe bei der Untersuchung gerade das Gefühl gehabt, dass sich etwas in meinem Kopf dreht.						
0<br>nein, gar nicht<br>○	1<br>sehr leicht<br>○	2<br>leicht<br>○	3<br>mittel<br>○	4<br>stark<br>○	5<br>sehr stark<br>○	weiß nicht<br>○

48) Bei meinem Schwindel(anfall) hat es sich …				
nicht in meinem Kopf gedreht<br>○	weniger in meinem Kopf gedreht als bei der Untersuchung<br>○	stärker in meinem Kopf gedreht als bei der Untersuchung<br>○	genauso stark in meinem Kopf gedreht wie bei der Untersuchung<br>○	weiß nicht<br>○

49) Ich habe bei der Untersuchung gerade irgendwie keine richtige Kontrolle mehr über meinen Körper gehabt.						
0<br>nein, gar nicht<br>○	1<br>sehr leicht<br>○	2<br>leicht<br>○	3<br>mittel<br>○	4<br>stark<br>○	5<br>sehr stark<br>○	weiß nicht<br>○

50) Bei meinem Schwindel(anfall) hatte ich…				
so ein Gefühl des Kontrollverlusts nicht<br>○	so ein Gefühl des Kontrollverlust weniger als bei der Untersuchung<br>○	so ein Gefühl des Kontrollverlusts stärker als bei der Untersuchung<br>○	so ein Gefühl des Kontrollverlusts genauso stark wie bei der Untersuchung<br>○	weiß nicht<br>○

51) Ich habe bei der Untersuchung gerade die Empfindung gehabt, dass ein Schwindel vom Nacken hochkommt.						
0<br>nein, gar nicht<br>◯	1<br>sehr leicht<br>◯	2<br>leicht<br>◯	3<br>mittel<br>◯	4<br>stark<br>◯	5<br>sehr stark<br>◯	weiß nicht<br>◯

52) Bei meinem Schwindel(anfall) hatte ich...				
so ein Gefühl nicht<br>◯	so ein Gefühl weniger als bei der Untersuchung<br>◯	so ein Gefühl stärker als bei der Untersuchung<br>◯	so ein Gefühl genauso stark wie bei der Untersuchung<br>◯	weiß nicht<br>◯

53) Ich habe bei der Untersuchung gerade das Gefühl gehabt, dass in meinem Kopf irgendwas nicht stimmt.						
0<br>nein, gar nicht<br>◯	1<br>sehr leicht<br>◯	2<br>leicht<br>◯	3<br>mittel<br>◯	4<br>stark<br>◯	5<br>sehr stark<br>◯	weiß nicht<br>◯

54) Bei meinem Schwindel(anfall) hatte ich ...				
nicht das Gefühl, dass in meinem Kopf etwas nicht stimmt<br>◯	das Gefühl, dass in meinem Kopf etwas nicht stimmt weniger als bei der Untersuchung<br>◯	das Gefühl, dass in meinem Kopf etwas nicht stimmt stärker als bei der Untersuchung<br>◯	das Gefühl, dass in meinem Kopf etwas nicht stimmt genauso stark wie bei der Untersuchung<br>◯	weiß nicht<br>◯

55) Ich habe bei der Untersuchung gerade das Gefühl gehabt, keinen klaren Gedanken fassen zu können, fast als ob mein Gehirn wie leergefegt wäre.						
0<br>nein, gar nicht<br>◯	1<br>sehr leicht<br>◯	2<br>leicht<br>◯	3<br>mittel<br>◯	4<br>stark<br>◯	5<br>sehr stark<br>◯	weiß nicht<br>◯

56) Bei meinem Schwindel(anfall) hatte ich ...				
so ein Gefühl der Leere im Kopf nicht<br>◯	so ein Gefühl der Leere im Kopf weniger als bei der Untersuchung<br>◯	so ein Gefühl der Leere im Kopf stärker als bei der Untersuchung<br>◯	so ein Gefühl der Leere im Kopf genauso stark wie bei der Untersuchung<br>◯	weiß nicht<br>◯

57) Ich habe bei der Untersuchung gerade den Eindruck gehabt, dass in meinem Kopf etwas hin und her schwappt.						
0<br>nein, gar nicht<br>◯	1<br>sehr leicht<br>◯	2<br>leicht<br>◯	3<br>mittel<br>◯	4<br>stark<br>◯	5<br>sehr stark<br>◯	weiß nicht<br>◯

58) Bei meinem Schwindel(anfall) hatte ich ...				
nicht das Gefühl, dass etwas in meinem Kopf hin und her schwappt<br>◯	so ein Gefühl weniger als bei der Untersuchung<br>◯	so ein Gefühl stärker als bei der Untersuchung<br>◯	so ein Gefühl genauso stark wie bei der Untersuchung<br>◯	weiß nicht<br>◯

59) Ich habe bei der Untersuchung gerade das Gefühl gehabt, dass sich Teile meines Körpers oder sogar mein ganzer Körper anders anfühlen (z.B. wie taub, pelzig oder kribbelig).						
0<br>nein, gar nicht<br>◯	1<br>sehr leicht<br>◯	2<br>leicht<br>◯	3<br>mittel<br>◯	4<br>stark<br>◯	5<br>sehr stark<br>◯	weiß nicht<br>◯

60) Bei meinem Schwindel(anfall) hatte ich...				
nicht so ein Gefühl, dass sich etwas anders anfühlt<br>◯	so ein Gefühl weniger als bei der Untersuchung<br>◯	so ein Gefühl stärker als bei der Untersuchung<br>◯	so ein Gefühl genauso stark wie bei der Untersuchung<br>◯	weiß nicht<br>◯

4c) Global item

Fragebogen Vergleich 7

61) Ich habe die Untersuchung insgesamt…				
ganz anders erlebt als meinen Schwindelanfall	eher anders erlebt als meinen Schwindelanfall	ähnlich erlebt wie meinen Schwindelanfall	fast gleich erlebt wie meinen Schwindelanfall	genau gleich erlebt wie meinen Schwindelanfall
○	○	○	○	○
